# Supplementary material for: Indications and endoscopic findings of upper gastrointestinal diseases in Africa: A systematic review & meta-analysis
Source: PLoS One. 2025 Mar 13;20(3):e0319854. doi: 10.1371/journal.pone.0319854 (PMC11906052; doi:10.1371/journal.pone.0319854)
Supplement: S1 Appendix — (DOCX) [file pone.0319854.s002.docx]

**S1 Appendix: Search strategies**

1. **Google Scholar:**

Search conducted was conducted up to December 31, 2024 where possible terms were searched in MeSH as well as text

Record Retrieved=1430

As sample we used to the following search strategies: Esophageal cancer, reflux esophagitis, eosinophilic esophagitis, esophageal varices, Barrett's esophagus, esophageal ulcers, and esophageal strictures. Gastric cancer, gastritis, gastric ulcers, gastric erosions, gastric polyps, gastric outlet obstruction, and pyloric stenosis. Duodenal cancer, duodenitis, duodenal ulcers, and duodenal erosions AND esophagogastroduodenoscopy," "upper GI endoscopy," and "gastrointestinal endoscopy AND [Countries name].

1. **Search Conducted in Hinari**

Search conducted up to December 31, 2024

Possible terms were searched in MeSH as well as text words

Result Retrieved: 465

Summon™: Esophageal cancer, reflux esophagitis, eosinophilic esophagitis, esophageal varices, Barrett's esophagus, esophageal ulcers, and esophageal strictures. Gastric cancer, gastritis, gastric ulcers, gastric erosions, gastric polyps, gastric outlet obstruction, and pyloric stenosis. Duodenal cancer, duodenitis, duodenal ulcers, and duodenal erosions AND esophagogastroduodenoscopy," "upper GI endoscopy," and "gastrointestinal endoscopy AND Africa.

**Search Conducted in Medline (PubMed)**

Search conducted up to December 31, 2024, where possible terms were searched in MeSH as well as text words

Record Retrieved=241

| #4 | (((((((((((((((((((((((Dyspepsia[Title/Abstract]) OR (Anorexia[Title/Abstract])) OR (Weight Loss[Title/Abstract])) OR (Odynophagia[Title/Abstract])) OR (Vomiting[Title/Abstract])) OR (Dysphagia[Title/Abstract])) OR (Anemia[Title/Abstract])) OR (Esophageal Varices[Title/Abstract])) OR (Upper GI Bleeding[Title/Abstract])) OR (Nausea[Title/Abstract])) OR (Esophageal Strictures[Title/Abstract])) OR (Peptic Ulcer Disease[Title/Abstract])) OR (Barrett's Esophagus[Title/Abstract])) OR (Achalasia[Title/Abstract])) OR (Persistent Bloating[Title/Abstract])) OR (Gastroduodenal Obstruction[Title/Abstract])) OR (Suspected Foreign Body[Title/Abstract])) OR (Caustic Ingestion[Title/Abstract])) OR (Portal Hypertension[Title/Abstract])) OR (Melena[Title/Abstract])) OR (Hematemesis[Title/Abstract])) OR ((((((((((((((((((((((((((Esophageal Cancer[Title/Abstract]) OR (Reflux Esophagitis[Title/Abstract])) OR (Eosinophilic Esophagitis[Title/Abstract])) OR (Peptic Esophagitis[Title/Abstract])) OR (Esophageal Cancer[Title/Abstract])) OR (Barrett's Esophagus[Title/Abstract])) OR (Esophageal Varices[Title/Abstract])) OR (Esophageal Strictures[Title/Abstract])) OR (Achalasia[Title/Abstract])) OR (Gastroesophageal Reflux Disease[Title/Abstract])) OR (Esophageal Ulcers[Title/Abstract])) OR (Esophageal Obstruction[Title/Abstract])) OR (Esophageal Foreign Body[Title/Abstract])) OR (Gastritis[Title/Abstract])) OR (Peptic Ulcer Disease[Title/Abstract])) OR (Gastric Cancer[Title/Abstract])) OR (Gastric Ulcers[Title/Abstract])) OR (Gastric Erosions[Title/Abstract])) OR (Gastric Polyps[Title/Abstract])) OR (Gastric Atrophy[Title/Abstract])) OR (Pyloric Stenosis[Title/Abstract])) OR (Duodenal Ulcer[Title/Abstract])) OR (Duodenitis[Title/Abstract])) OR (Duodenal Cancer[Title/Abstract])) OR (Duodenal Erosions[Title/Abstract])) OR (Gastric Outlet Obstruction[Title/Abstract]))) AND ((((Esophagogastroduodenoscopy[Title/Abstract]) OR (Endoscopy[Title/Abstract])) OR (Gastrointestinal Endoscopy[Title/Abstract])) OR (Upper GI Endoscopy[Title/Abstract]))) AND ((((((((((((((((((((((((((((((((((((((((((((((((((((((Algeria[Title/Abstract]) OR (Angola[Title/Abstract])) OR (Benin[Title/Abstract])) OR (Botswana[Title/Abstract])) OR (Burkina Faso[Title/Abstract])) OR (Burundi[Title/Abstract])) OR (Cabo Verde[Title/Abstract])) OR (Cameroon[Title/Abstract])) OR (Central African Republic[Title/Abstract])) OR (Chad[Title/Abstract])) OR (Comoros[Title/Abstract])) OR (Democratic Republic of the Congo[Title/Abstract])) OR (Republic of the Congo[Title/Abstract])) OR (Cote d'Ivoire[Title/Abstract])) OR (Djibouti[Title/Abstract])) OR (Egypt[Title/Abstract])) OR (Equatorial Guinea[Title/Abstract])) OR (Eritrea[Title/Abstract])) OR (Eswatini[Title/Abstract])) OR (Ethiopia[Title/Abstract])) OR (Gabon[Title/Abstract])) OR (Gambia[Title/Abstract])) OR (Ghana[Title/Abstract])) OR (Guinea[Title/Abstract])) OR (Guinea-Bissau[Title/Abstract])) OR (Kenya[Title/Abstract])) OR (Lesotho[Title/Abstract])) OR (Liberia[Title/Abstract])) OR (Libya[Title/Abstract])) OR (Madagascar[Title/Abstract])) OR (Malawi[Title/Abstract])) OR (Mali[Title/Abstract])) OR (Mauritania[Title/Abstract])) OR (Mauritius[Title/Abstract])) OR (Morocco[Title/Abstract])) OR (Mozambique[Title/Abstract])) OR (Namibia[Title/Abstract])) OR (Niger[Title/Abstract])) OR (Nigeria[Title/Abstract])) OR (Rwanda[Title/Abstract])) OR (Sao Tome[Title/Abstract] AND Principe[Title/Abstract])) OR (Senegal[Title/Abstract])) OR (Seychelles[Title/Abstract])) OR (Sierra Leone[Title/Abstract])) OR (Somalia[Title/Abstract])) OR (South Africa[Title/Abstract])) OR (South Sudan[Title/Abstract])) OR (Sudan[Title/Abstract])) OR (Tanzania[Title/Abstract])) OR (Togo[Title/Abstract])) OR (Tunisia[Title/Abstract])) OR (Uganda[Title/Abstract])) OR (Zambia[Title/Abstract])) OR (Zimbabwe[Title/Abstract])) | 241 |
| --- | --- | --- |
| #3 | (((((((((((((((((((((((((((((((((((((((((((((((((((((Algeria[Title/Abstract]) OR (Angola[Title/Abstract])) OR (Benin[Title/Abstract])) OR (Botswana[Title/Abstract])) OR (Burkina Faso[Title/Abstract])) OR (Burundi[Title/Abstract])) OR (Cabo Verde[Title/Abstract])) OR (Cameroon[Title/Abstract])) OR (Central African Republic[Title/Abstract])) OR (Chad[Title/Abstract])) OR (Comoros[Title/Abstract])) OR (Democratic Republic of the Congo[Title/Abstract])) OR (Republic of the Congo[Title/Abstract])) OR (Cote d'Ivoire[Title/Abstract])) OR (Djibouti[Title/Abstract])) OR (Egypt[Title/Abstract])) OR (Equatorial Guinea[Title/Abstract])) OR (Eritrea[Title/Abstract])) OR (Eswatini[Title/Abstract])) OR (Ethiopia[Title/Abstract])) OR (Gabon[Title/Abstract])) OR (Gambia[Title/Abstract])) OR (Ghana[Title/Abstract])) OR (Guinea[Title/Abstract])) OR (Guinea-Bissau[Title/Abstract])) OR (Kenya[Title/Abstract])) OR (Lesotho[Title/Abstract])) OR (Liberia[Title/Abstract])) OR (Libya[Title/Abstract])) OR (Madagascar[Title/Abstract])) OR (Malawi[Title/Abstract])) OR (Mali[Title/Abstract])) OR (Mauritania[Title/Abstract])) OR (Mauritius[Title/Abstract])) OR (Morocco[Title/Abstract])) OR (Mozambique[Title/Abstract])) OR (Namibia[Title/Abstract])) OR (Niger[Title/Abstract])) OR (Nigeria[Title/Abstract])) OR (Rwanda[Title/Abstract])) OR (Sao Tome[Title/Abstract] AND Principe[Title/Abstract])) OR (Senegal[Title/Abstract])) OR (Seychelles[Title/Abstract])) OR (Sierra Leone[Title/Abstract])) OR (Somalia[Title/Abstract])) OR (South Africa[Title/Abstract])) OR (South Sudan[Title/Abstract])) OR (Sudan[Title/Abstract])) OR (Tanzania[Title/Abstract])) OR (Togo[Title/Abstract])) OR (Tunisia[Title/Abstract])) OR (Uganda[Title/Abstract])) OR (Zambia[Title/Abstract])) OR (Zimbabwe[Title/Abstract]) |  |
| #2 | (((Esophagogastroduodenoscopy[Title/Abstract]) OR (Endoscopy[Title/Abstract])) OR (Gastrointestinal Endoscopy[Title/Abstract])) OR (Upper GI Endoscopy[Title/Abstract]) | 95989 |
| #1 | (((((((((((((((((((((((((Esophageal Cancer[Title/Abstract]) OR (Reflux Esophagitis[Title/Abstract])) OR (Eosinophilic Esophagitis[Title/Abstract])) OR (Peptic Esophagitis[Title/Abstract])) OR (Esophageal Cancer[Title/Abstract])) OR (Barrett's Esophagus[Title/Abstract])) OR (Esophageal Varices[Title/Abstract])) OR (Esophageal Strictures[Title/Abstract])) OR (Achalasia[Title/Abstract])) OR (Gastroesophageal Reflux Disease[Title/Abstract])) OR (Esophageal Ulcers[Title/Abstract])) OR (Esophageal Obstruction[Title/Abstract])) OR (Esophageal Foreign Body[Title/Abstract])) OR (Gastritis[Title/Abstract])) OR (Peptic Ulcer Disease[Title/Abstract])) OR (Gastric Cancer[Title/Abstract])) OR (Gastric Ulcers[Title/Abstract])) OR (Gastric Erosions[Title/Abstract])) OR (Gastric Polyps[Title/Abstract])) OR (Gastric Atrophy[Title/Abstract])) OR (Pyloric Stenosis[Title/Abstract])) OR (Duodenal Ulcer[Title/Abstract])) OR (Duodenitis[Title/Abstract])) OR (Duodenal Cancer[Title/Abstract])) OR (Duodenal Erosions[Title/Abstract])) OR (Gastric Outlet Obstruction[Title/Abstract]) | 206,987 |
