# Supplementary material for: Indications and endoscopic findings of upper gastrointestinal diseases in Africa: A systematic review & meta-analysis
Source: PLoS One. 2025 Mar 13;20(3):e0319854. doi: 10.1371/journal.pone.0319854 (PMC11906052; doi:10.1371/journal.pone.0319854)
Supplement: S2 Appendix — (DOCX) [file pone.0319854.s003.docx]

**S2 Appendix. Quality assessment of included studies.**

**Reviewer**: Seid Mohammed Abdu and Hussen Abdu, Date: May 12, 2024

**Author**:

**Year**: 2024.

Yes No Unclear N/A

1. Was the sample frame appropriate

to address the target population? □ □ □

1. Were study participant’s sample

in an appropriate way? □ □ □

1. Was the sample size adequate? □ □ □
2. Were the study subjects and

the setting described in detail? □ □ □

1. Was the data analysis conducted with

sufficient coverage of the identified sample?   □ □ □

1. Were valid methods used for

the identification of the condition?   □ □ □

1. Was the condition measured in

a standard, reliable way for all participants? □ □ □

1. Was there appropriate statistical analysis   □ □ □
2. Was the response rate adequate, and if not, was

the low response rate managed appropriately? □ □ □

Based on these criteria, the quality score for the prevalence study was given in the following table.

**Table S1**. JBI Critical Appraisal Checklist for included Prevalence Studies

| **Author Name, (publication Yr)** | Criteria and corresponding scores | | | | | | | | | | | | | Overall score |
| --- | --- | --- | --- | --- | --- | --- | --- | --- | --- | --- | --- | --- | --- | --- |
|  | **#1** | | **#2** | **#3** | **#4** | **#5** | **#6** | | **#7** | | **#8** | | **#9** |  |
| Argaw, A.M., et al. 2023[1] | 1 | | 1 | 1 | 1 | 1 | 1 | | 1 | | 1 | | 1 | 9 |
| Assefa, B., et al. 2022[2] | 1 | | 1 | 1 | 1 | 1 | 1 | | 1 | | 1 | | 1 | 9 |
| Melak W, et al. 2023[3] | 1 | | 1 | 1 | 1 | 1 | 1 | | 1 | | 1 | | 1 | 7 |
| Kiros YK et al.2017 [4] | 1 | | 1 | 1 | 0 | 1 | 1 | | 1 | | 1 | | 1 | 8 |
| Getahun GM et al. (2015[5] | 1 | | 1 | 0 | 1 | 1 | 1 | | 1 | | 1 | | 1 | 8 |
| Zena D et al. 2024[6] | 1 | | 1 | 1 | 1 | 1 | 1 | | 1 | | 1 | | 1 | 9 |
| Makanga W, et al. 2014 [7] | 1 | | 1 | 1 | 1 | 1 | 1 | | 1 | | 1 | | 1 | 9 |
| Mwangi CN. et al. 2020[8] | 1 | | 1 | 1 | 1 | 1 | 1 | | 1 | | 1 | | 1 | 9 |
| Ayuo PO, et al. 2014[9] | 1 | | 1 | 1 | 1 | 1 | 1 | | 1 | | 1 | | 1 | 9 |
| Lodenyo H. et al. 2005[10] | 1 | | 1 | 1 | 0 | 1 | 1 | | 1 | | 1 | | 1 | 8 |
| Adani AA et al. 2023[11] | 1 | | 1 | 1 | 1 | 1 | 1 | | 1 | | 1 | | 1 | 9 |
| Bulur O et al. 2018[12] | 1 | | 1 | 1 | 0 | 1 | 1 | | 1 | | 1 | | 1 | 8 |
| Obayo S. et al. 2015[13] | 0 | | 0 | 1 | 1 | 0 | 1 | | 1 | | 1 | | 1 | 6 |
| Namugerwa J. et al. 2017[14] | 0 | | 0 | 0 | 1 | 0 | 1 | | 1 | | 1 | | 1 | 5 |
| Okello TR, et al. (2016)[15] | 1 | | 1 | 1 | 1 | 1 | 1 | | 1 | | 1 | | 1 | 9 |
| Abeshouse MA, et al. 2024[16] | 0 | | 0 | 0 | 1 | 1 | 1 | | 1 | | 1 | | 1 | 6 |
| Doe MJ et al.(2021)[17] | 0 | | 0 | 1 | 1 | 0 | 1 | | 1 | | 1 | | 1 | 5 |
| Walker TD et al 2014 [18] | 1 | | 1 | 1 | 0 | 1 | 1 | | 1 | | 1 | | 1 | 6 |
| Ayana SM et al. 2014[19] | 1 | | 1 | 1 | 0 | 1 | 1 | | 1 | | 1 | | 1 | 7 |
| Qu LS, et al. 2023 [20] | 1 | | 0 | 1 | 1 | 1 | 1 | | 1 | | 1 | | 1 | 8 |
| Khamisi R H. 2013[21] | 1 | | 1 | 1 | 1 | 0 | 1 | | 1 | | 1 | | 1 | 7 |
| Said EM et al. 2014[22] | 0 | | 0 | 0 | 0 | 0 | 1 | | 1 | | 1 | | 1 | 4 |
| El Shallaly et al.2021[23] | 1 | | 1 | 1 | 1 | 0 | 1 | | 1 | | 1 | | 1 | 8 |
| Elhadi AA et al. 2014[24] | 1 | | 0 | 1 | 0 | 1 | 1 | | 1 | | 1 | | 1 | 8 |
| Adam HY et al. 2008[25] | 1 | | 0 | 1 | 0 | 1 | 1 | | 1 | | 1 | | 1 | 8 |
| Yahya H. 2023[26] | 1 | | 1 | 1 | 1 | 0 | 1 | | 1 | | 1 | | 0 | 8 |
| Ray-Offor E. et al. 2020[27] | 1 | 1 | | 1 | 1 | 1 | 1 | 1 | | 1 | | 1 | | 9 |
| Okoye OG. et al.2021 [28] | 1 | | 0 | 0 | 0 | 1 | 1 | | 1 | | 1 | | 1 | 6 |
| Odeghe E A. et al. 2023[29] | 1 | | 1 | 1 | 0 | 1 | 1 | | 1 | | 1 | | 1 | 8 |
| Obonna GC et al. 2020[30] | 0 | | 0 | 0 | 1 | 1 | 1 | | 1 | | 1 | | 1 | 6 |
| Ismaila BO. et al. 2013[31] | 0 | | 0 | 0 | 0 | 1 | 1 | | 1 | | 1 | | 1 | 5 |
| Misauno M. et al. 2011[32] | 0 | | 1 | 1 | 1 | 1 | 1 | | 1 | | 1 | | 1 | 8 |
| Ngim O et al. 2017 [33] | 1 | | 0 | 1 | 1 | 1 | 1 | | 1 | | 1 | | 1 | 8 |
| Jeje EA et al. 2013[34] | 1 | | 1 | 1 | 0 | 1 | 1 | | 1 | | 1 | | 1 | 8 |
| Nwokediuko SC et al. 2012[35] | 1 | | 1 | 1 | 1 | 0 | 1 | | 1 | | 1 | | 1 | 8 |
| Oluwagbenga OO et al[36] | 0 | | 0 | 0 | 1 | 1 | 1 | | 1 | | 1 | | 1 | 6 |
| Archampong TN. et al.2016 [37] | 1 | | 1 | 1 | 1 | 0 | 1 | | 1 | | 1 | | 1 | 8 |
| Darko R et al 2015[38] | 1 | | 1 | 1 | 1 | 0 | 1 | | 1 | | 1 | | 1 | 8 |
| Agyei-NkansahA et al. 2019[39] | 1 | | 1 | 1 | 0 | 1 | 1 | | 1 | | 1 | | 1 | 8 |
| Duah A et al.2022[40] | 1 | | 1 | 1 | 1 | 1 | 1 | | 1 | | 1 | | 1 | 9 |
| Aduful HK. et al. 2007[41] | 1 | | 1 | 1 | 1 | 1 | 1 | | 1 | | 1 | | 1 | 9 |
| Gyedu A, and Yorke J 2014[42] | 1 | | 1 | 1 | 0 | 1 | 1 | | 1 | | 1 | | 1 | 8 |
| Dakubo JC et al. 2011[43] | 1 | | 1 | 1 | 0 | 1 | 1 | | 1 | | 1 | | 1 | 8 |
| Tabiri S et al.2015[44] | 1 | | 1 | 1 | 0 | 1 | 1 | | 1 | | 1 | | 1 | 8 |
| Koura M. et al.2017[45] | 1 | | 1 | 1 | 1 | 1 | 1 | | 1 | | 1 | | 1 | 9 |
| Meda ZC et al. 2023[46] | 1 | | 1 | 1 | 1 | 1 | 1 | | 1 | | 1 | | 1 | 9 |
| Okon JB et al. 2021[47] | 1 | | 1 | 1 | 1 | 0 | 1 | | 1 | | 1 | | 1 | 8 |
| Gado A. et al. 2015[48] | 1 | | 1 | 1 | 1 | 1 | 1 | | 1 | | 1 | | 1 | 8 |
| El-Ghannam R et al. 2019 [49] | 0 | | 0 | 0 | 0 | 0 | 1 | | 1 | | 1 | | 1 | 4 |
| Gomaa AA et al. 2022 [50] | 1 | | 1 | 1 | 1 | 1 | 1 | | 1 | | 1 | | 1 | 9 |
| Elbadry M et al. 2024 [51] | 1 | | 1 | 1 | 1 | 1 | 1 | | 1 | | 1 | | 1 | 9 |
| Abdelrazek FG et al. 2024 [52] | 1 | | 1 | 1 | 1 | 1 | 1 | | 1 | | 1 | | 1 | 9 |
| Raafat KM. et al 2022 [53] | 0 | | 0 | 0 | 1 | 1 | 1 | | 1 | | 1 | | 1 | 6 |
| Yasser MY et al. 2023[54] | 1 | | 1 | 1 | 1 | 1 | 1 | | 1 | | 1 | | 1 | 9 |
| Moustafa HM, et al. 2023[55] | 1 | | 1 | 1 | 1 | 1 | 1 | | 1 | | 1 | | 1 | 9 |
| Fouad M et al. 2018[56] | 1 | | 1 | 1 | 1 | 1 | 1 | | 1 | | 1 | | 1 | 9 |
| Ali MH et al. 2024 [57] | 1 | | 1 | 1 | 1 | 1 | 1 | | 1 | | 1 | | 1 | 9 |
| Tumi A. et al.2007[58] | 0 | | 0 | 0 | 0 | 0 | 1 | | 1 | | 1 | | 1 | 4 |
| Cheddie S.et al.2020 [59] | 1 | | 1 | 1 | 1 | 1 | 1 | | 1 | | 1 | | 1 | 9 |
| Mnyombolo Y et al. 2022[60] | 1 | | 1 | 1 | 1 | 1 | 1 | | 1 | | 1 | | 1 | 9 |
| Ntola VC et al. 2019[61] | 0 | | 0 | 0 | 0 | 1 | 1 | | 1 | | 1 | | 1 | 5 |
| [Fernando N et al.](https://journals.asm.org/doi/full/10.1128/jcm.39.4.1323-1327.2001#con1) 2001[62] | 0 | | 1 | 0 | 0 | 1 | 1 | | 1 | | 1 | | 1 | 6 |
| Kayamba V, et al. [63] | 1 | | 1 | 1 | 1 | 1 | 1 | | 1 | | 1 | | 1 | 9 |
| Kelly P et al.2008[64] | 1 | | 1 | 1 | 1 | 1 | 1 | | 1 | | 1 | | 1 | 9 |
| Kayamba V. et al 2015 [65] | 1 | | 1 | 1 | 1 | 1 | 1 | | 1 | | 1 | | 1 | 9 |
| Wolf LL. et al. 2012[66] | 1 | | 1 | 1 | 1 | 1 | 1 | | 1 | | 1 | | 1 | 9 |
| Mothes H. et al. 2009[67] | 1 | | 1 | 1 | 1 | 1 | 1 | | 1 | | 1 | | 1 | 9 |
| Adonis NM et al.2021 [68] | 1 | | 1 | 1 | 1 | 1 | 1 | | 1 | | 1 | | 1 | 9 |

**NB**: 1 indicates the article does fulfill the specified criteria

0 indicates the article does not fulfill the stated criteria

1. Argaw, A.M., et al., *Indications and Findings of Upper Gastrointestinal Endoscopy at a Tertiary Hospital in Ethiopia: A Cross-Sectional Study.* Clinical and Experimental Gastroenterology, 2023: p. 187-196.

2. Assefa, B., et al., *Peptic ulcer disease among dyspeptic patients at endoscopy unit, University of Gondar hospital, Northwest Ethiopia.* BMC gastroenterology, 2022. **22**(1): p. 164.

3. Melak, W., et al., *Predictive value of alarm features in diagnosing upper gastrointestinal malignancies among dyspeptic patients: A cross-sectional study in Ethiopia.* Gastroenterol Hepatol Res, 2023. **5**(3): p. 13.

4. Kiros, Y.K., B. Tsegay, and H. Abreha, *Endoscopic and histopathological correlation of gastrointestinal diseases in ayder referral hospital, Mekelle University, Northern Ethiopia.* Ethiopian Medical Journal, 2017. **55**(4).

5. Getahun, G.M. and Z.A. Abubeker, *UPPER GASTROINTESTINAL ENDOSCOPY FINDINGS AT GONDAR UNIVERSITY HOSPITAL, NORTH-WESTERN ETHIOPIA: AN EIGHT YEAR ANALYSIS.* International journal of Pharmaceuticals and Health care research **Vol.03, No. 2)**: p. 60-65.

6. Zena, D., et al., *Patterns of upper gastrointestinal diseases among patients undergoing esophagogastroduodenoscopy at three hospitals in Asella town, Southeast Ethiopia.* Scientific Reports, 2024. **14**(1): p. 24067.

7. Makanga, W. and A. Nyaoncha, *Upper gastrointestinal disease in Nairobi and Nakuru counties, Kenya; a two year comparative endoscopy study.* Annals of African Surgery, 2014. **11**(2).

8. Mwangi, C.N., et al., *Prevalence and endoscopic findings of Helicobacter pylori infection among dyspeptic patients in Kenya.* Open Journal of Medical Microbiology, 2020. **10**(04): p. 233.

9. Ayuo, P., F. Some, and J. Kiplagat, *Upper gastrointestinal endoscopy findings in patients referred with upper gastrointestinal symptoms in Eldoret, Kenya: a retrospective review.* East African Medical Journal, 2014. **91**(8): p. 267-273.

10. Lodenyo, H., et al., *Patterns of upper gastrointestinal diseases based on endoscopy in the period 1998-2001.* African Journal of Health Sciences, 2005. **12**(1): p. 49-54.

11. Adani, A.A., et al., *Helicobacter pylori status and associated upper gastrointestinal endoscopic diagnosis in a tertiary hospital: A retrospective study.* Journal of Clinical Sciences, 2023. **20**(4): p. 118-122.

12. Bulur, O., et al., *The only and first analysis of upper gastrointestinal endoscopy results from Mogadishu-Somalia.* Turkiye Klinikleri Cardiovascular Sciences, 2018. **30**(1): p. 1-5.

13. Obayo, S., et al., *Upper gastrointestinal diseases in patients for endoscopy in South-Western Uganda.* Afr Health Sci, 2015. **15**(3): p. 959-66.

14. Namugerwa, J., *Peptic ulcer prevalence among patient attending Kampala International University Teaching Hospital in Ishaka Bushenyi Municipality.* 2017.

15. Okello, T., D. Ogwang, and I. Pecorella, *An evaluation of 605 endoscopic examination in a rural setting, lacor hospital in northern Uganda.* British Journal of Medicine and Medical Research, 2016. **15**(10).

16. Abeshouse, M.A.M., et al., *The impact of introducing The impact of introducing diagnostic and therapeutic upper endoscopy in an ambulatory Surgery Center in Rural Eastern Uganda diagnostic and therapeutic upper endoscopy in an ambulatory Surgery Center in Rural Eastern Uganda.* African Health Sciences, 2024. **24**(2): p. 437-444.

17. Doe, M.J., et al., *Upper gastrointestinal endoscopy findings in Mbale Regional Referral Hospital, Eastern Uganda: a 10-year retrospective analysis.* African Health Sciences, 2021. **21**(2): p. 919-926.

18. Walker, T.D., et al., *Helicobacter pylori status and associated gastroscopic diagnoses in a tertiary hospital endoscopy population in Rwanda.* Transactions of the Royal Society of Tropical Medicine and Hygiene, 2014. **108**(5): p. 305-307.

19. Ayana, S.M., et al., *Upper gastrointestinal endoscopic findings and prevalence of Helicobacter pylori infection among adult patients with dyspepsia in northern Tanzania.* Tanzania journal of health research, 2014. **16**(1).

20. Qu, L.-S. and M.M. Gubi, *Clinical features of upper gastrointestinal endoscopy in 3146 patients: a 9-year retrospective cohort study in Zanzibar Archipelago, Tanzania.* African Health Sciences, 2023. **23**(2): p. 393-401.

21. Khamisi, R., *Indications and findings of patients undergoing upper gastrointestinal endoscopy at Muhimbili National Hospital, Dar es salaam, Tanzania*. 2013, Muhimbili University of Health and Allied Sciences.

22. Said, E.M., et al., *A New Horizon for Gastrointestinal Endoscopy in Port Sudan, Sudan: Through Concept, Design and Delivery? A Visiting Practitioner’s Commentary.* Global Journal of Gastroenterology & Hepatology, 2014. **2**(1): p. 35-40.

23. El Shallaly, G.E.H.A., et al., *The Change in Upper Gastrointestinal Disease Pattern in Sudan.* Dysphagia, 2021. **123**: p. 6.8.

24. Elhadi, A., et al., *Pattern of endoscopic findings of upper gastrointestinal tract in Omdurman teaching hospital, Sudan.* Sudan Journal of Medical Sciences, 2014. **9**(2): p. 71-74.

25. Adam, H.Y. and E. Doumi, *Upper Gastrointestinal Endoscopy in El Obeid, Western Sudan: Analysis of the First 1150 Cases.* Sudan Journal of Medical Sciences, 2008. **3**(2): p. 91-94.

26. Yahya, H., *Change in prevalence and pattern of peptic ulcer disease in the Northern Savannah of Nigeria: An endoscopic study.* Annals of African Medicine, 2023. **22**(4): p. 420-425.

27. Ray-Offor, E. and K.A. Opusunju, *Current status of peptic ulcer disease in Port Harcourt metropolis, Nigeria.* African Health Sciences, 2020. **20**(3): p. 1446-1451.

28. Okoye, O.G., et al., *Research Article Correlation of Clinical, Endoscopic, and Pathological Findings among Suspected Peptic Ulcer Disease Patients in Abuja, Nigeria.* 2021.

29. Odeghe, E.A., et al., *Appropriateness and diagnostic yield of open access gastroscopy in two tertiary centers in South-western Nigeria.* African Health Sciences, 2023. **23**(2): p. 386-92.

30. Obonna, G. and M. Obonna, *Gastrointestinal Endoscopy in the Riverine Southwestern Ondo State of Nigeria: An Eight Year Review.* Western Journal of Medical and Biomedical Sciences, 2020. **1**(1): p. 81-88.

31. Ismaila, B.O. and M.A. Misauno, *Gastrointestinal endoscopy in Nigeria-a prospective two year audit.* Pan African Medical Journal, 2013. **14**(1).

32. Misauno, M., et al., *SPECTRUM OF ENDOSCOPICALLY DIAGNOSED UPPER GASTROINTESTINAL DISEASES IN JOS.* Sahel Medical Journal, 2011. **14**(2): p. 63-66.

33. Ngim, O., et al., *A Two Year Review of Upper Gastrointestinal Endoscopy in Calabar, Nigeria.* IOSR J Med Dent Sci, 2017. **16**: p. 31-4.

34. Jeje, E., T. Olajide, and B. Akande, *Upper gastrointestinal endoscopy-our findings, our experience in Lagoon Hospital, Lagos, Nigeria.* 2013.

35. Nwokediuko, S.C., et al., *Time trends of upper gastrointestinal diseases in Nigeria.* Annals of gastroenterology, 2012. **25**(1): p. 52.

36. Oluwagbenga, O.O., et al., *Upper gastrointestinal endoscopy in Ido-ekiti, Nigeria: a four-year review.* Open Journal of Gastroenterology and Hepatology, 2020. **3**(2): p. 35-35.

37. Archampong, T.N., et al., *Factors associated with gastro-duodenal disease in patients undergoing upper GI endoscopy at the Korle-Bu Teaching Hospital, Accra, Ghana.* Afr Health Sci, 2016. **16**(2): p. 611-9.

38. Darko, R., et al., *Changing patterns of the prevalence of Helicobacter pylori among patients at a corporate hospital in Ghana.* Ghana Medical Journal, 2015. **49**(3): p. 147-153.

39. Agyei-Nkansah, A., A. Duah, and M. Alfonso, *Indications and findings of upper gastrointestinal endoscopy in patients presenting to a District Hospital, Ghana.* Pan African Medical Journal, 2019. **34**(1).

40. Duah, A., et al., *Indications and findings of oesophagogastroduodenoscopy in patients with symptoms of upper gastrointestinal disease in Eastern Regional Hospital, Koforidua, Ghana.* PAMJ Clinical Medicine, 2022. **10**(18).

41. Aduful, H., et al., *Upper gastrointestinal endoscopy at the korle bu teaching hospital, accra, ghana.* Ghana Med J, 2007. **41**(1): p. 12-6.

42. Gyedu, A. and J. Yorke, *Upper gastrointestinal endoscopy in the patient population of Kumasi, Ghana: indications and findings.* The Pan African Medical Journal, 2014. **18**.

43. Dakubo, J., J. Clegg-Lamptey, and P. Sowah, *Appropriateness of referrals for upper gastrointestinal endoscopy.* West African journal of medicine, 2011. **30**(5): p. 342-347.

44. Tabiri, S., A. Prosper, and A. Adam, *Upper gastrointestinal endoscopic findings in patients presenting to Tamale Teaching Hospital, Ghana.* Unified Journal of Medicine and Medical Sciences, 2015. **Vol 1(2)**: p. 006- 011.

45. Koura, M., et al., *Upper gastrointestinal endoscopy at University Hospital Souro Sanou Bobo-Dioulasso (Burkina Faso), about 1022 cases: signs and lesions observed.* Open Journal of Gastroenterology, 2017. **7**(11): p. 287-296.

46. Ziemle Clement, M., et al., *Acceptability of Oesogastroduodenal Fibroscopy in Private Health Facilities from the City of Bobo-Dioulasso in Burkina Faso.* Central African Journal of Public Health, 2023.

47. Okon, J.B., et al., *Factors Associated with the Applicability of EPAGE (European Panel on the Appropriateness of Gastrointestinal Endoscopy) and the Suitability of Indications for Eso-Gastroduodenal Endoscopy in a West African Country.* Open Journal of Gastroenterology, 2021. **11**(10): p. 173-183.

48. Gado, A., et al., *Endoscopic evaluation of patients with dyspepsia in a secondary referral hospital in Egypt.* Alexandria Journal of Medicine, 2015. **51**(3): p. 179–184-179–184.

49. El-Ghannam, R., et al., *Endoscopic and Microbiological Findings of Helicobacter pylori Infection among Dyspeptic Patients in Suez Canal University Hospital.* Egyptian Journal of Medical Microbiology, 2019. **28**(4): p. 103-109.

50. Gomaa, A.A., E.A. Hassan, and A.Y. El-sary, *Cross section study of endoscopic findings in patients underwent upper endoscopy in Fayoum University Hospital.* Fayoum University Medical Journal, 2022. **10**(1): p. 7-17.

51. Elbadry, M., et al., *Clinical and endoscopic characteristics of patients undergoing gastrointestinal endoscopic procedures in Egypt: a nationwide multicenter study.* BMC Gastroenterology, 2024. **24**(1): p. 186.

52. Abdelrazek, F.G., et al., *Clinico-Endoscopic profile of Egyptian Patients presenting with Upper Gastrointestinal Symptoms.* Al-Azhar International Medical Journal, 2024. **5**(6): p. 44.

53. Raafat, K.M., et al., *Correlation between Gastrointestinal Symptoms Questionnaire and Findings of Upper Gastrointestinal Endoscopy in Gastrointestinal Disorders.* The Egyptian Journal of Hospital Medicine, 2022. **88**(1): p. 2339-2548.

54. Yasser, M.Y., et al., *Pattern and Risk Factors of Upper Gastrointestinal Endoscopy Associated Bacterial Infections in Suez Canal University.* Egyptian Journal of Medical Microbiology, 2023. **32**(2): p. 89-93.

55. Moustafa, H.M., et al., *Upper endoscopic findings in patients attending the endoscopy unit of al-azhar assiut university hospital: 2019–2020.* Al-Azhar Assiut Medical Journal, 2023. **21**(2): p. 110-117.

56. Fouad, M., et al., *Prevalence of eosinophilic esophagitis in adult patients with upper gastrointestinal symptoms in a locality in upper Egypt.* Clinical Endoscopy, 2018. **51**(4): p. 357-361.

57. Hussein Mohammed, A., A.M. Zaghloul, and M. Malak, *Study of upper gastrointestinal endoscopic patterns among patients who underwent Esophagogastroduodenoscopy in Sohag University Hospital.* Sohag Medical Journal, 2024. **28**(3): p. 42-50.

58. Tumi, A., et al., *Prevalence of Helicobacter pylori infection in patients with dyspepsia in Tripoli central hospital, Tripoli, Libya.* Libyan J Inf Dis, 2007. **1**: p. 124-7.

59. Cheddie, S., C. Manneh, and Y. Moodley, *Alarm features as predictors of major findings in a rural South African upper endoscopic service.* South African Journal of Surgery, 2020. **58**(4): p. 216a-216e.

60. Mnyombolo, Y. and S. Pillay, *Indications, outcomes and complications of endoscopies performed at a regional hospital in Kwazulu-Natal, South Africa.* South African Gastroenterology Review, 2022. **20**(1): p. 32-40.

61. Ntola, V., et al., *An audit of upper gastrointestinal endoscopy performed on patients at Prince Mshiyeni Memorial Hospital in Durban, KwaZulu-Natal.* South African Journal of Surgery, 2019. **57**(3): p. 55-58.

62. Fernando, N., et al., *Helicobacter pylori infection in an urban African population.* Journal of clinical microbiology, 2001. **39**(4): p. 1323-1327.

63. Kayamba, V., M. Mubbunu, and P. Kelly, *Endoscopic diagnosis of gastric and oesophageal cancer in Lusaka, Zambia: a retrospective analysis.* BMC gastroenterology, 2024. **24**(1): p. 122.

64. Kelly, P., et al., *Gastrointestinal pathology in the University Teaching Hospital, Lusaka, Zambia: review of endoscopic and pathology records.* Transactions of the Royal society of Tropical Medicine and Hygiene, 2008. **102**(2): p. 194-199.

65. Kayamba, V., et al., *Trends in upper gastrointestinal diagnosis over four decades in Lusaka, Zambia: a retrospective analysis of endoscopic findings.* BMC gastroenterology, 2015. **15**: p. 1-9.

66. Wolf, L.L., et al., *Esophagogastroduodenoscopy in a public referral hospital in Lilongwe, Malawi: spectrum of disease and associated risk factors.* World J Surg, 2012. **36**(5): p. 1074-1082.

67. Mothes, H., et al., *Do patients in rural Malawi benefit from upper gastrointestinal endoscopy?* Tropical doctor, 2009. **39**(2): p. 73-76.

68. Adonis, N.M., et al., *Profile of Endoscopic Lesions and Prevalence of H. pylori Infection at the Digestive Endoscopy Unit of Panzi General Reference Hospital in Bukavu.* Open Journal of Gastroenterology, 2021. **11**(11): p. 230-243.
