# Supplementary material for: Indications and endoscopic findings of upper gastrointestinal diseases in Africa: A systematic review & meta-analysis
Source: PLoS One. 2025 Mar 13;20(3):e0319854. doi: 10.1371/journal.pone.0319854 (PMC11906052; doi:10.1371/journal.pone.0319854)
Supplement: S1 Fig — (DOCX) [file pone.0319854.s004.docx]

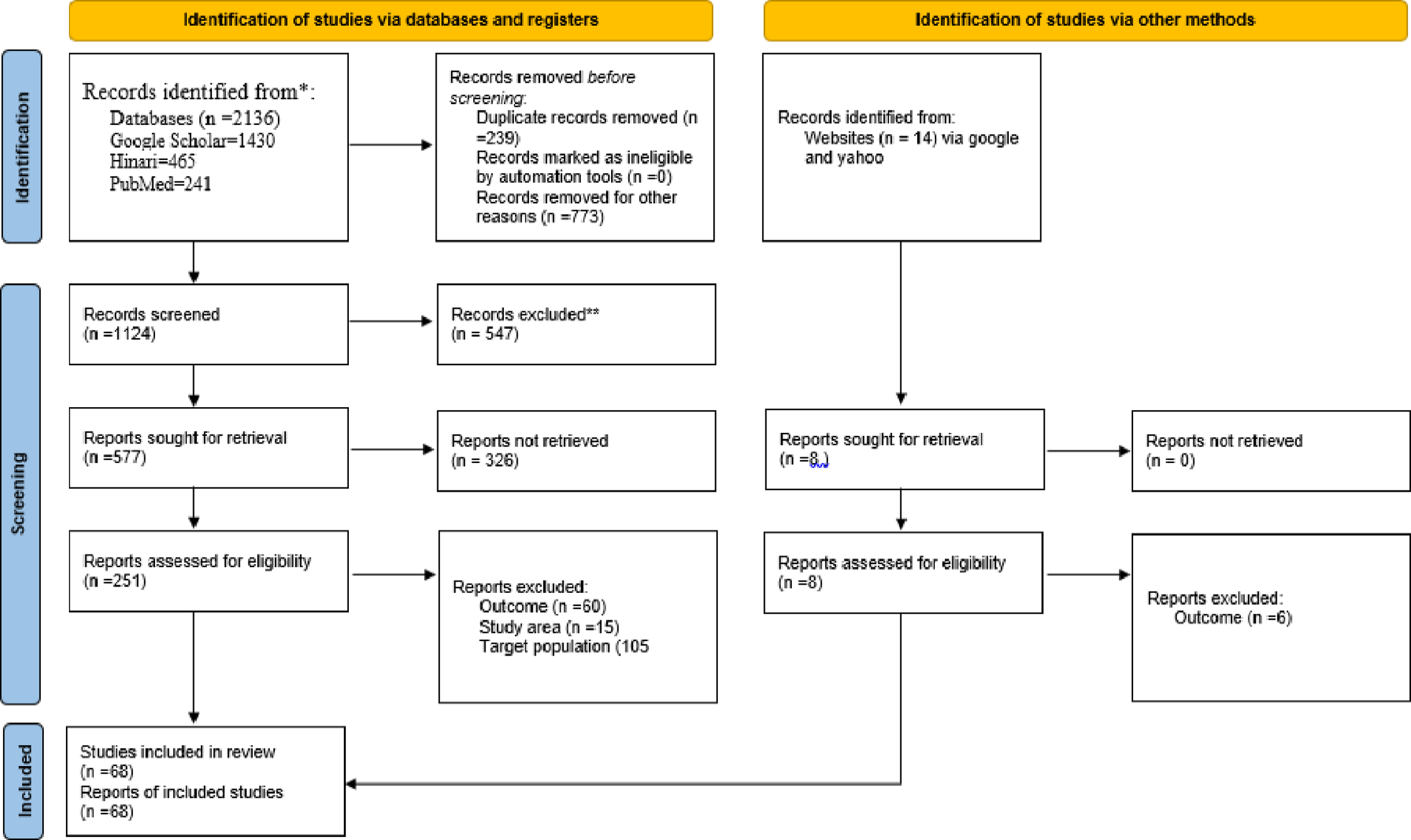


**S1 Fig: Depicts the schematic flow of study selection steps for indications and endoscopic findings of UGID in Africa**
