# Supplementary figures and images for: Indications and endoscopic findings of upper gastrointestinal diseases in Africa: A systematic review & meta-analysis
Source: PLoS One. 2025 Mar 13;20(3):e0319854. doi: 10.1371/journal.pone.0319854 (PMC11906052; doi:10.1371/journal.pone.0319854)

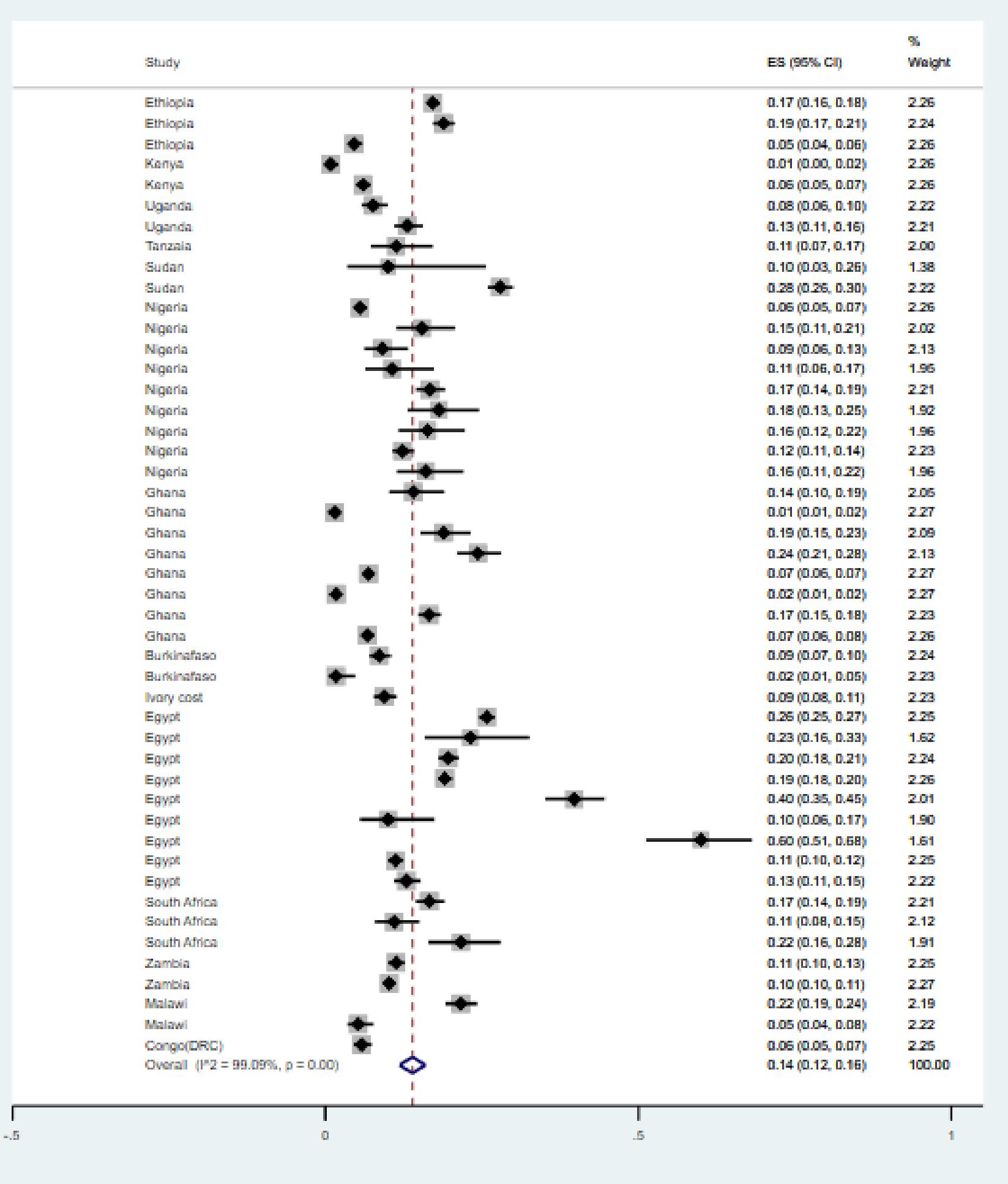


S2 Fig: Forest Plot of hematemesis as an Indication for Upper Gastrointestinal Endoscopy

Supplement: S2 Fig — (DOCX) [file pone.0319854.s005.docx]

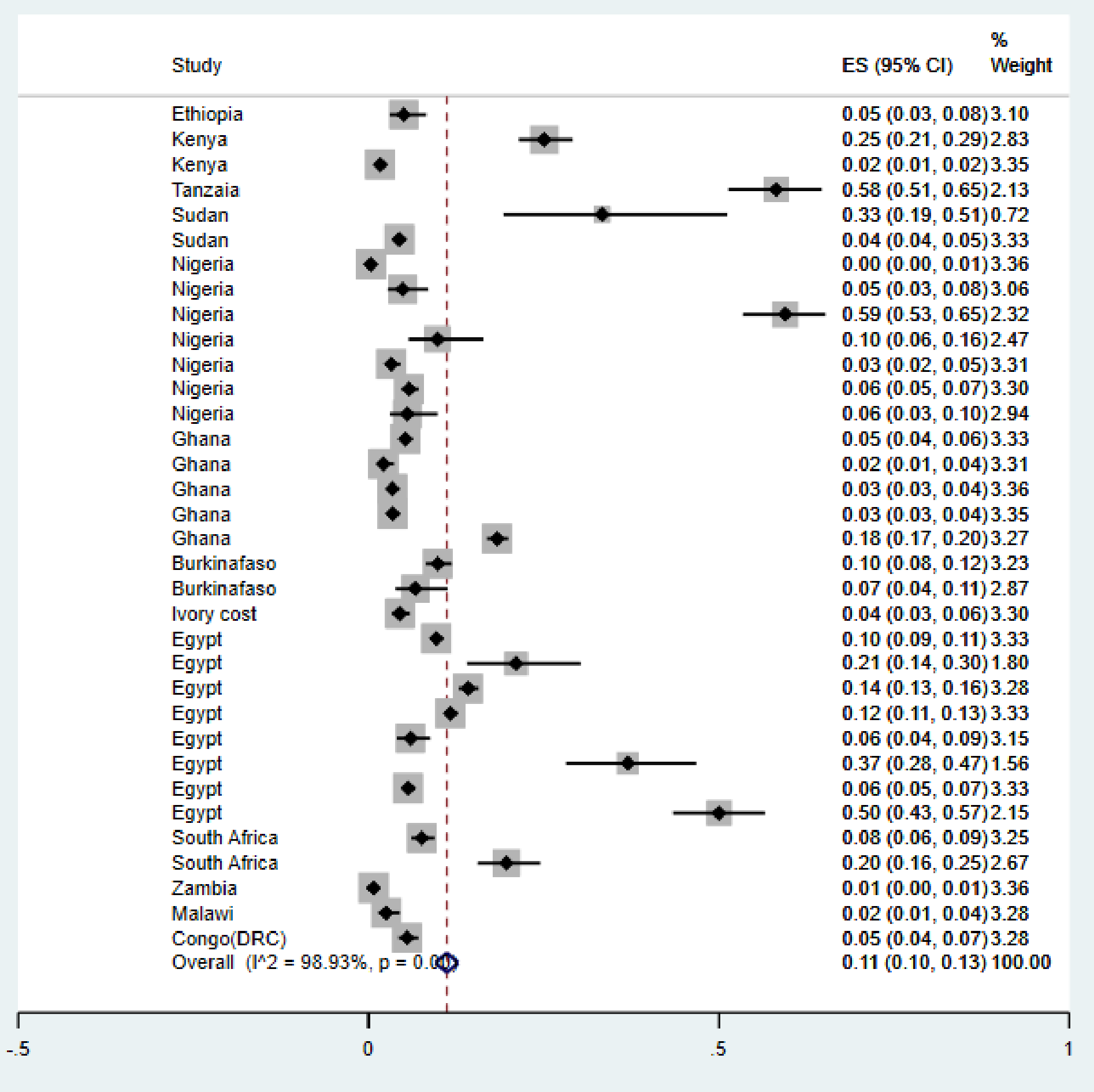


S3 Fig: Forest Plot of GERD symptoms as an Indication for Upper Gastrointestinal Endoscopy (UGIE)

Supplement: S3 Fig — (DOCX) [file pone.0319854.s006.docx]

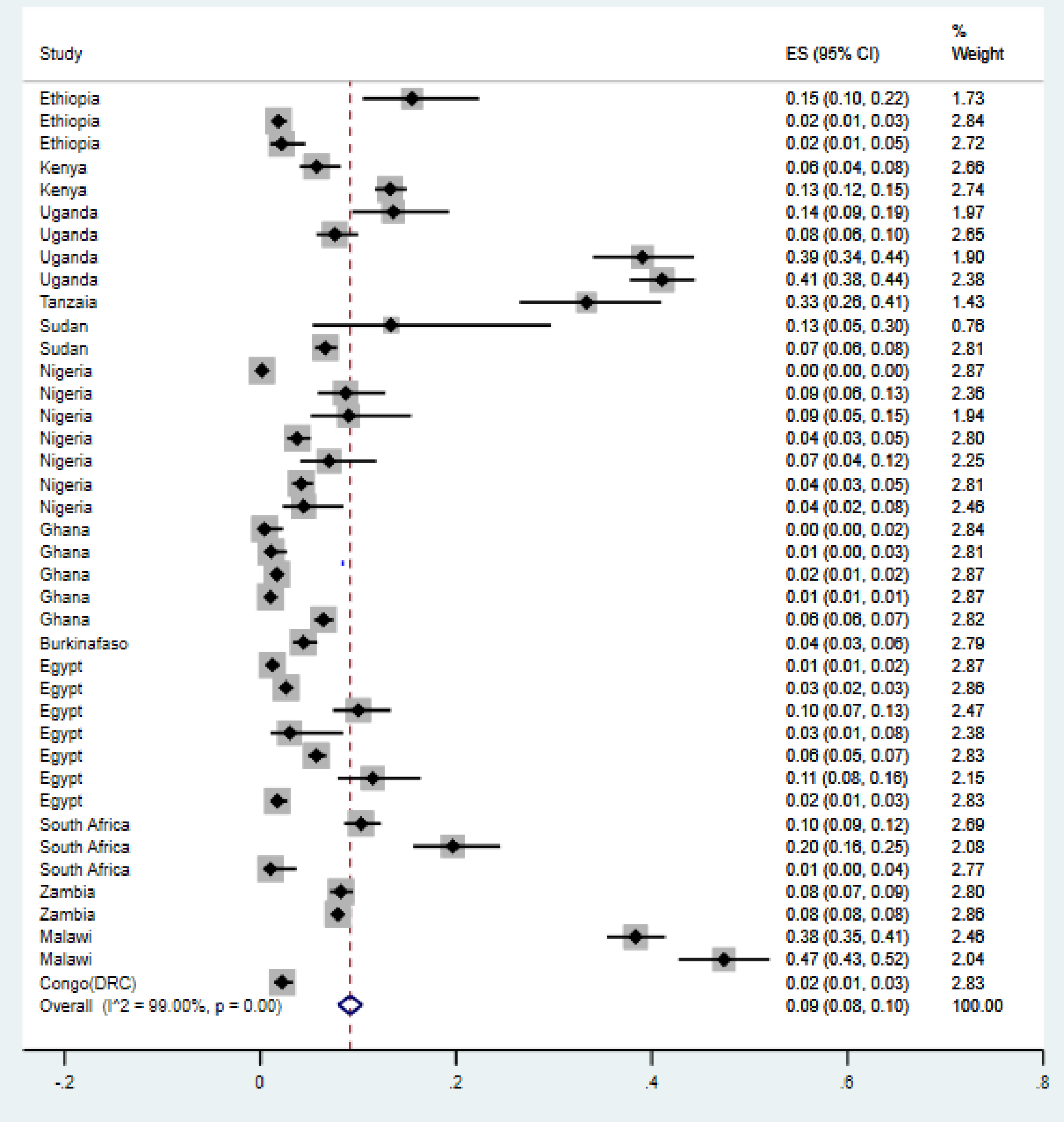


S4 Fig: Forest Plot of Dysphagia as an Indication for Upper Gastrointestinal Endoscopy (UGIE)

Supplement: S4 Fig — (DOCX) [file pone.0319854.s007.docx]

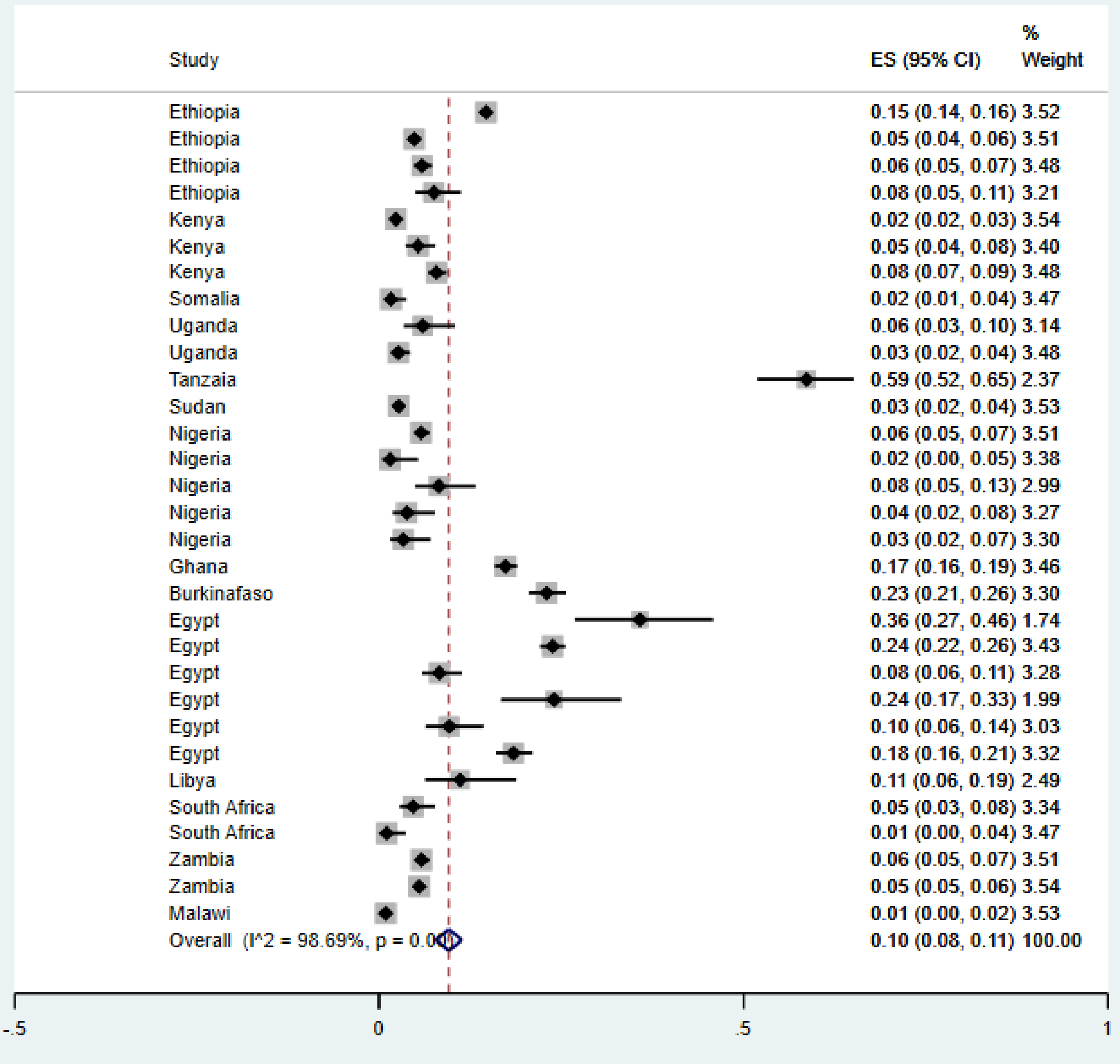


S5 Fig: Forest Plot of GERD as diagnosis of Upper Gastrointestinal Endoscopy (UGIE)

Supplement: S5 Fig — (DOCX) [file pone.0319854.s008.docx]

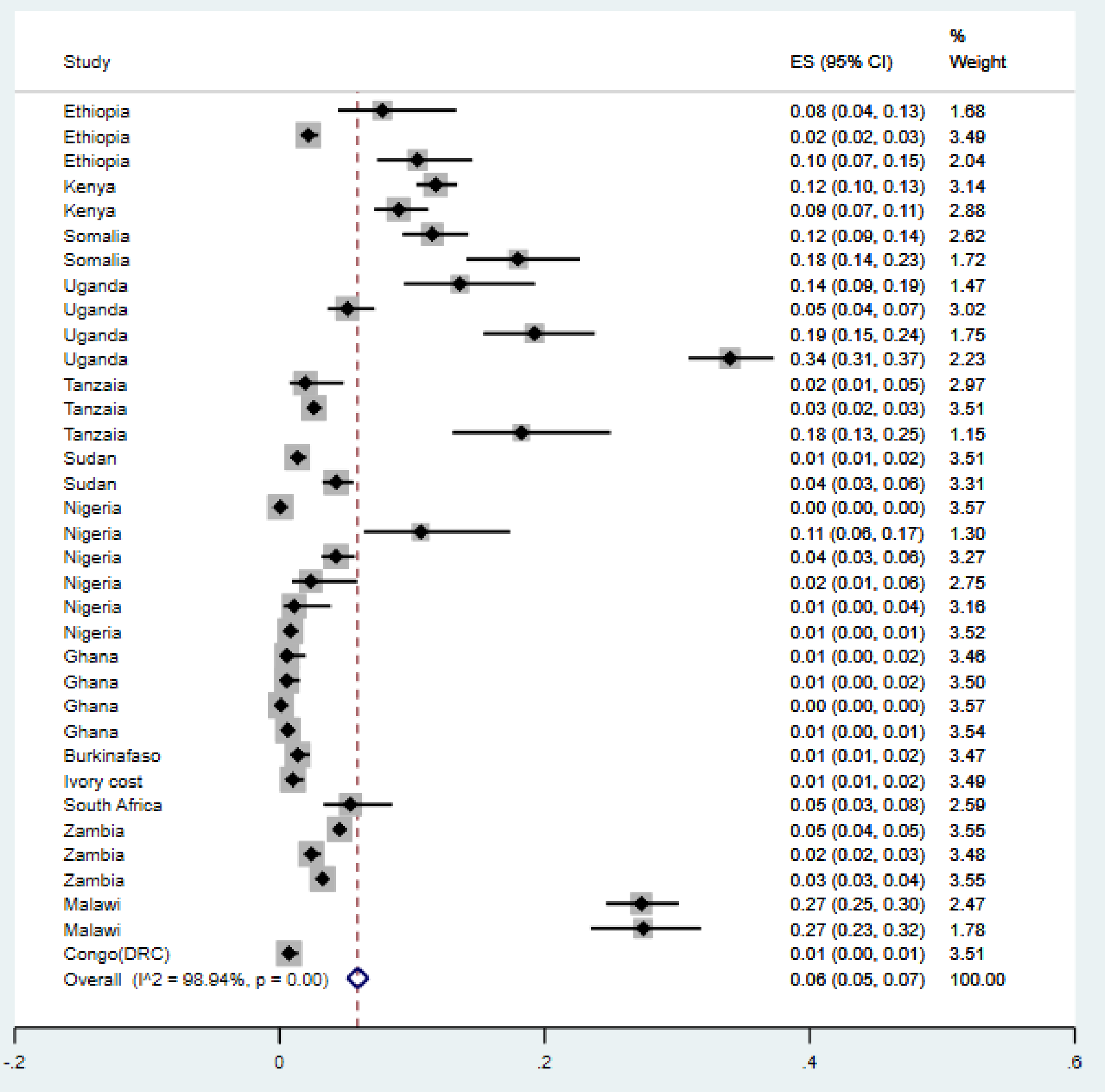


S6 Fig: Forest Plot of Esophageal cancer as diagnosis of Upper Gastrointestinal Endoscopy (UGIE)

Supplement: S6 Fig — (DOCX) [file pone.0319854.s009.docx]
