# Supplementary material for: Indications and endoscopic findings of upper gastrointestinal diseases in Africa: A systematic review & meta-analysis
Source: PLoS One. 2025 Mar 13;20(3):e0319854. doi: 10.1371/journal.pone.0319854 (PMC11906052; doi:10.1371/journal.pone.0319854)
Supplement: Attachment — Submitted filename: Response to Rivewers (1).docx [file pone.0319854.s010.docx]

**A Rebuttal Letter**

Dear esteemed editor **Dr. Muhammad Salman Bashir** and reviewers,

We greatly value your feedback and comments provided on our manuscript titled "Indications and Endoscopic Findings of Upper Gastrointestinal Diseases in Africa: A Systematic Review & Meta-analysis". All of the points that you raised are important, improve the quality of the work, and contain details that the journal requires.

Dear, we made every effort to answer every one of your remarks. The responses that follow have been made in response to each one. Please find below a point-by-point response to all comments and concerns raised. Reviewer comments are shown in black, followed by our corresponding responses in dark blue. Changes to the manuscript have been highlighted in blue in the revised version for easy access. We want to thank you again for your wonderful feedback and appreciate any more suggestions or remarks you may have to help us improve the work.

Best regards,

A point-by-point response to the comments given

A. **Response to Comments of the Academic Editor**

**Comment #1:** Dear Dr. Abdu,

Thank you for submitting your manuscript to PLOS ONE. After careful consideration, we feel that it has merit but does not fully meet PLOS ONE’s publication criteria as it currently stands. Therefore, we invite you to submit a revised version of the manuscript that addresses the points raised in the review process.
Please include the following items when submitting your revised manuscript:

- A rebuttal letter that responds to each point raised by the academic editor and reviewer(s). You should upload this letter as a separate file labeled 'Response to Reviewers'.
- A marked-up copy of your manuscript that highlights changes made to the original version. You should upload this as a separate file labeled 'Revised Manuscript with Track Changes'.
- An unmarked version of your revised paper without tracked changes. You should upload this as a separate file labeled 'Manuscript'.

If you would like to make changes to your financial disclosure, please include your updated statement in your cover letter. Guidelines for resubmitting your figure files are available below the reviewer comments at the end of this letter.
If applicable, we recommend that you deposit your laboratory protocols in [protocols.io](http://protocols.io/) to enhance the reproducibility of your results.

Journal requirements:

When submitting your revision, we need you to address these additional requirements.

1. Please ensure that your manuscript meets PLOS ONE's style requirements, including those for file naming.

**Response #1:** Dear editor, we agree with your suggestion and we have tried to incorporate all of your concerns throughout the revised manuscript. We also tried to adjust the manuscript to follow the requirements of PLOS ONE's style.

**Comment #2**. We note that your Data Availability Statement is currently as follows: [All relevant data are within the manuscript and its Supporting Information files]

Please confirm at this time whether or not your submission contains all the raw data required to replicate the results of your study. Authors must share the “minimal data set” for their submission. PLOS defines the minimal data set to consist of the data required to replicate all study findings reported in the article, as well as related metadata and methods. For example, authors should submit the following data:

- The values behind the means, standard deviations, and other measures reported;

- The values used to build graphs;

- The points extracted from images for analysis.

Authors do not need to submit their entire data set if only a portion of the data was used in the reported study. If your submission does not contain these data, please either upload them as Supporting Information files or deposit them to a stable, public repository and provide us with the relevant URLs, DOIs, or accession numbers.

**Response #2:** Thank you for your inquiry regarding the Data Availability Statement.

We confirm that our submission contains the necessary information to replicate the results of our study. We have provided the **raw data used for the meta-analysis** as a supplementary Excel file **(S2 Appendix)**. This file includes the relevant metadata extracted during the analysis, which are required to replicate the findings reported in the manuscript. However, we would like to clarify that we do not have the raw quantitative data (e.g., individual data points or values behind the means and standard deviations).

In accordance with PLOS's definition of the minimal data set, we believe that the information provided in the Excel file is sufficient for the replication of our findings.

Furthermore, we have included sample forest plots in the Supporting Information section of this revision, which displays the results presented in the tables. So, we believe all necessary data required to replicate our study findings are available within the manuscript and Supporting Information.

If you require any additional details or specific data points, please let us know, and we will do our best to accommodate your request.

**Comment #3:** If there are ethical or legal restrictions on sharing a de-identified data set, please explain them in detail (e.g., data contain potentially sensitive information, data are owned by a third-party organization, etc.) and who has imposed them (e.g., an ethics committee). Please also provide contact information for a data access committee, ethics committee, or other institutional body to which data requests may be sent. If data are owned by a third party, please indicate how others may request data access.

**Response #3**: Our study is a systematic review and meta-analysis, and all data included in the analysis were derived from publicly available sources, specifically, previously published studies. As no primary data were collected, there are no ethical or legal restrictions on sharing a de-identified data set. The data used are already in the public domain, and thus, no specific ethical approval or restrictions apply to the sharing of this data.

**Comment #4:** As required by our policy on Data Availability, please ensure your manuscript or supplementary information includes the following: A numbered table of all studies identified in the literature search, including those that were excluded from the analyses. For every excluded study, the table should list the reason(s) for exclusion.

**Response #4**: Thank you for your comment regarding the request for a numbered table of all studies identified in the literature search, including those excluded from the analyses.

During the data extraction process, we carefully followed the inclusion and exclusion criteria as outlined in the methodology. In total, we identified 2,850 studies, of which 48 were included in the final analysis, as reflected in the PRISMA flow chart.

The remaining 2,802 studies were excluded based on various criteria, including irrelevance to the topic, duplication, inadequate methodology, or failure to meet the inclusion criteria. These excluded studies were deleted during the data extraction process, and as a result, we do not have a complete list of their citations. However, the exclusion process was meticulously followed and is clearly documented in the PRISMA flow diagram, which details the screening steps and reasons for exclusion.

**Practical Limitations:**

Given the large number of excluded studies (2,802), providing a full list of their titles would be impractical at this stage. However, the reasons for their exclusion are transparently outlined in the PRISMA flow chart, ensuring the rigor of the screening process.

**Search Strategy Transparency:**

As a sample of our approach, we have provided the full PubMed search strategy in the supplementary materials, which can be used to replicate the initial search and screening process. Similar strategies were applied across other databases, including Google Scholar, HINARI, and general web searches (e.g., Google and Yahoo) to capture gray literature. However, it is important to note that search platforms like Google Scholar and general web search engines do not allow for precise search replication due to their evolving algorithms and limited filtering capabilities. Despite these challenges, we applied the same key search terms and inclusion/exclusion criteria across all platforms, as outlined in our methodology.

We hope this explanation provides clarity, and we are happy to provide further details on our methodology or search strategy if needed

**Comment #5:** If any of the included studies are unpublished, include a link (URL) to the primary source or detailed information about how the content can be accessed.

**Response #5**: Thank you for your query. We would like to clarify that no unpublished studies were included in our systematic review and meta-analysis.

**Comment #6:** A table of all data extracted from the primary research sources for the systematic review and/or meta-analysis. The table must include the following information for each study: Name of data extractors and date of data extraction. Confirmation that the study was eligible to be included in the review. All data extracted from each study for the reported systematic review and/or meta-analysis that would be needed to replicate your analyses.

**Response #6**: Thank you for your comment regarding the data extraction table. As requested, we have included a detailed table in the Supporting Information file of the revised manuscript ( S2 Appendix).

**Comment #7:** If data or supporting information were obtained from another source (e.g. correspondence with the author of the original research article), please provide the source of data and dates on which the data/information were obtained by your research group.

**Response #7:** Thank you for your concern regarding the source of data and supporting information. For our systematic review and meta-analysis, all data were extracted directly from the original published studies included in our analysis. We did not obtain any additional data or supporting information from other sources, such as correspondence with the authors of the original research articles. If any data had been obtained through direct communication with the authors, we would have provided the appropriate sources and dates in the manuscript. However, this did not apply to our study.

**Comment #8:** If applicable for your analysis, a table showing the completed risk of bias and quality/certainty assessments for each study or outcome.  Please ensure this is provided for each domain or parameter assessed. For example, if you used the Cochrane risk-of-bias tool for randomized trials, provide answers to each of the signaling questions for each study. If you used GRADE to assess the certainty of the evidence, provide judgments about each of the quality of evidence factors. This should be provided for each outcome.

**Response #8**: Thank you for your comment regarding the risk of bias and quality/certainty assessments. For our systematic review and meta-analysis, we assessed the risk of bias and quality of included studies using the Joanna Briggs Institute (JBI) tool, as appropriate for the types of studies analyzed. We have provided a table in the Supporting Information file that includes the completed risk of bias and quality assessments for each study, covering all relevant domains and parameters assessed using the JBI tool. This table contains specific details for each domain and parameter, ensuring full transparency in the evaluation of each study.

**Comment #9:** An explanation of how missing data were handled.

**Response #9**: Thank you for your comment regarding the handling of missing data. In our systematic review and meta-analysis, we focused on upper gastrointestinal (UGI) endoscopy indications reported in at least two or more studies. If a study did not report a particular indication, we interpreted this as a potential absence of data for that specific indication, rather than as missing data. To ensure robust analyses, we included only the indications that were consistently reported in two or more studies. If a study lacked data on a particular indication, it was excluded from that specific analysis but still included in others where it met the reporting criteria. This approach allowed us to focus on well-reported indications while maintaining the integrity of our meta-analysis.

**B. Response to Comments of the Reviewers**

Review Comments to the Author
Please use the space provided to explain your answers to the questions above. You may also include additional comments for the author, including concerns about dual publication, research ethics, or publication ethics. (Please upload your review as an attachment if it exceeds 20,000 characters)

**Response to Comments of the Reviewer #1**

**Comment #1:** Potential Factual Inaccuracy. The paper states that "to the best of our knowledge, there has been no systematic assessment of endoscopic indications and findings of upper GI disease in Africa." However, similar reports already exist, suggesting a potential factual inaccuracy. Specifically, the 2019 paper by Timothy N. Archampong et al., titled "Gastro-duodenal disease in Africa: Literature review and clinical data from Accra, Ghana," provides data on the prevalence of PUD and H. pylori infection. This report emphasizes the need to consider H. pylori in the current study's analysis, particularly because NSAID use was identified as a possible reason for the persistently high prevalence of PUD in some African countries.

**Response #1:** Thank you for raising this concern. We would like to clarify that there is no factual inaccuracy in the statement. The paper by Timothy N. Archampong et al. (2019) is **a literature review** that focuses primarily on peptic ulcer disease (PUD), its pattern, and gastric histopathology across Africa. Their study does not provide a systematic review and meta-analysis of all endoscopic indications and findings, it narrows its focus to peptic ulcer disease (PUD) and related histopathological features. They included 9 studies from both Africa and outside Africa to discuss PUD and its patterns, and 17 studies to explore the histopathology of PUD. In contrast, our study is a systematic review and meta-analysis that aims to provide a comprehensive evaluation of the most common indications for upper gastrointestinal endoscopy (UGIE) and to summarize the overall endoscopic findings related to upper gastrointestinal diseases (UGID) across Africa only. We synthesized data from 48 studies, identifying a wide range of indications (such as epigastric pain and dyspepsia) and endoscopic findings (such as gastritis, functional dyspepsia, and GERD). Our work offers a broader and more systematic assessment of UGID across the continent only, making it distinct from the **literature review** by Archampong et al. Therefore, there is no factual inaccuracy in our statement, as our study represents the first comprehensive systematic review and meta-analysis on this specific topic.

**Comment #2:** Limitations in Search Methodology

Despite the search period extending up to 2024, important recent studies are missing from the analysis. For example, the 2023 study titled "Indications and Findings of Upper Gastrointestinal Endoscopy at a Tertiary Hospital in Ethiopia: A Cross-Sectional Study" was not included in the results. This raises concerns about the quality of the search strategy or hand search, suggesting that other significant papers may also have been overlooked.

**Response #2:** Thank you for your valuable feedback. We acknowledge that the 2023 study titled "Indications and Findings of Upper Gastrointestinal Endoscopy at a Tertiary Hospital in Ethiopia: A Cross-Sectional Study" was inadvertently omitted from Table 1 in our manuscript. However, the study findings were indeed included in our analysis. We have now corrected this oversight by updating Table 1 to ensure that the study is properly listed among the included articles. We apologize for this error and have revised the table to accurately reflect the studies used in our analysis.

Upon further review of the included studies, we discovered that a study from Burkina Faso, conducted by Coulibaly et al., was inadvertently included in our presentation despite not meeting our inclusion criteria. While this study was excluded from our analysis, it was mistakenly listed in Table 1, taking the position of the Ethiopian study. The Ethiopian study was included in the analysis, but due to this oversight, it was not reflected correctly in the table. We have now corrected this error by removing the Burkina Faso study from the table and reinstating the Ethiopian study in its rightful position. Consequently, the total number of studies included remains 48, and the findings from the Ethiopian study have been fully accounted for in the analysis.

We appreciate the reviewer’s concern regarding the thoroughness of our search strategy. We would like to emphasize that we conducted a comprehensive search and hand search to capture all relevant studies. The issue with the Ethiopian study was not a failure of the search strategy but an inadvertent omission from the table. We have now rectified this, and we will conduct additional verification to ensure that no further significant studies were overlooked.
**Comment #3:** Inadequate Search Results
The search string provided in the paper only resulted in finding an older study (a 1994 paper on prescriptions for peptic ulcer drugs in Iceland), which suggests a problem with the effectiveness of the search strategy. This raises concerns about whether the search strategy was able to capture a comprehensive set of relevant literature.

**Response #3:** Thank you for your valuable feedback. We acknowledge your concern regarding the search strategy, and we would like to clarify and provide additional information to address it.

**Clarification on the Search String in the Method Section:** The search string included in the original submission was a partial and simplified version meant to illustrate our approach to identifying relevant studies. Its purpose was to provide an overview of the search process, rather than presenting the full, detailed search strategy. We understand how this partial search string may have led to the identification of an older study (a 1994 paper on prescriptions for peptic ulcer drugs in Iceland), which may not appear directly relevant to the focus of our study. However, this was an isolated outcome of the search, and the full search strategy was more comprehensive and specific. To provide transparency and further demonstrate the robustness of our search process, we have now included the full search strategy used in this study, which covers all databases and search terms applied. This has been submitted as supporting information to ensure that the review process benefits from the complete details of our search methodology.

The full search strategy reflects a comprehensive approach, designed to capture all relevant studies on upper gastrointestinal diseases in Africa, including more recent publications and geographically relevant data. As mentioned in our previous response, we have successfully identified and included recent studies, such as the 2023 Ethiopian study, which is now correctly reflected in the updated version of the manuscript. The inclusion of older studies such as the 1994 Icelandic paper w
**Comment #4:** Lack of Data Availability.
The paper mentions that "all relevant data are within the manuscript and its Supporting Information files," but no data or links were provided, making it impossible to verify the findings. This lack of data transparency could impact the reproducibility and credibility of the study.
**Response #4:** Thank you for highlighting this issue. We sincerely apologize for the oversight in the initial submission regarding the absence of the supporting data files. We have corrected this issue, and all relevant data, including tables, figures, and supplementary materials, have been provided as **Supporting Information** in the updated submission. This includes all the necessary data files required to verify the findings of our systematic review and meta-analysis. And we understand the importance of data transparency and reproducibility in ensuring the credibility of scientific research. Therefore, we have made sure that the supporting files contain all datasets, search strategies, and additional details used in the analysis to facilitate independent verification.

**Comment #5:** Concerns Regarding Data Bias and Representativeness. The exclusion of data in native languages may have introduced bias in the analysis.

**Response #5:** Thank you for pointing out these important concerns regarding potential bias and the representativeness of our findings. We acknowledge the potential for bias due to the exclusion of studies published in native languages other than English. Our search strategy included English-language studies, which may have omitted relevant research published in other languages. However, our decision to focus on English-language articles was primarily driven by the accessibility and consistency of quality assessment tools available in English that have provided supporting information. While this may limit the scope of the study, it remains in line with standard practices in systematic reviews. We agree that including studies in native languages could have added further depth, and we will consider this in future analyses.

**Comment #6:** Additionally, the paper does not provide information on the overall number of upper GI endoscopy cases in Africa or how the current report represents a percentage of that total, raising concerns about the representativeness of the study. Specific data on these points would help in assessing the study's generalizability.

**Response #6:** Regarding the representativeness of our data, we would like to clarify that the included studies cover a wide geographical area across Africa, with diverse patient populations and clinical settings. This diversity helps to mitigate the risk of selection bias and enhances the generalizability of our findings. However, we recognize that we did not estimate the overall number of upper GI endoscopy (UGIE) cases across Africa. While there is no centralized registry or comprehensive data source for all UGIE cases across Africa, our analysis is based on available published studies. It thus reflects a subset of total UGIE cases. To strengthen the context of representativeness, we have now included additional discussion in the revised manuscript, acknowledging this limitation and explaining how the analyzed data fit within the larger context of UGIE practices in Africa.

**Response to Comments of the Reviewer #2**

**Comment #1:** The researchers conducted a systematic review and meta-analysis entitled “Indications and Endoscopic Findings of Upper Gastrointestinal Diseases in Africa: A Systematic Review & Meta-analysis. The review and meta-analysis are well-designed and conducted. The manuscript is well written. However, several points could be improved in the manuscript. The author reported as a primary indication: epigastric pain (50%), dyspepsia (43.7%), and abdominal pain (29.6%), only these 3 indications reached 123.3%. please clarify and explain the sum of more than 100%.

**Response #1:** Thank you so much for your encouragement and positive evaluation of our study design and manuscript. We appreciate your detailed feedback and suggestions for improvement. We appreciate the opportunity to clarify that the percentage reported for the primary indications (epigastric pain, dyspepsia, and abdominal pain) was pooled from different numbers of studies, not from the same dataset or patient. Specifically. Epigastric pain (50%) was reported in 19 studies, dyspepsia (43.7%) was reported in 14 studies, and abdominal pain (29.6%) was reported in 13 studies. Since these indications were extracted from different subsets of the total 48 studies, the reported percentages are relative to the patient population within these respective studies, not cumulative across all 48 studies. Thus, the sum of the percentages exceeds 100% because they are derived from different sample sizes, with patients in different studies presenting with different primary indications.

**Comment #2:** Moreover, epigastric pain, dyspepsia, and abdominal pain could be used for upper abdominal pain. Please explain the differences between the different indications as used in the studies and ensure that more than one of these indications is not used in the same patients (maybe the patients had dyspepsia and abdominal pain as indications)

**Response #2**: Thank you for highlighting this important concern. The data regarding these indications was extracted directly from the studies, each of which typically identified a single primary indication for each patient. Specifically, epigastric pain was reported in 19 studies, dyspepsia in 14 studies, and abdominal pain in 13 studies. Notably, most studies that reported dyspepsia did not also include abdominal pain, and the same pattern applies to the other indications. This approach significantly reduces the likelihood of double-counting or overlap in the pooled results. Furthermore, to maintain consistency, we adhered to the original terminology and classifications used in the research publications, without reclassifying or merging these indications into broader categories like "upper abdominal pain." This method ensures the integrity of the data collection and presentation.

**Comment #3:** Functional dyspepsia affected 29.8% of patients. Please explain how wad functional dyspepsia was diagnosed in EGD. Functional dyspepsia is diagnosed according to clinical criteria and not according to EGD.

**Response #3:** Thank you for your insightful question regarding the diagnosis of functional dyspepsia (FD) in our analysis. In the studies included in our systematic review, functional dyspepsia was primarily diagnosed based on clinical criteria and served as a diagnosis of exclusion following upper gastrointestinal endoscopy (EGD). Specifically, FD was identified in a patient who underwent EGD and was found to have no organic finding to explain their symptom. Out of the 32 reviewed studies, several reported that functional dyspepsia was diagnosed after ruling out structural abnormalities such as ulcers, gastritis, or malignancies during endoscopy. As a result, we classified these cases as functional dyspepsia based on the absence of identifiable organic causes. This diagnostic approach is consistent with current clinical guidelines, which recommend using endoscopy to exclude organic disease in patients presenting with dyspeptic symptoms.

**Comment #4:** Table 1: Reference number 50: please add the name of the author

**Response #4:** Thank you for pointing out this oversight. We have now updated Table 1 by including the same of the author for reference number 50, as requested. The table has been corrected accordingly.
**Comment #5:** Abbreviations: should explained in the first appearance in the text and below the tables, for example, table 3.

**Response #5:** Thank you for your valuable comment. We have ensured that all abbreviations are now explained at their first appearance in the text as well as below the relevant tables, including Table 3. This improves clarity and consistency throughout the manuscript.
**Comment #6:** What about Helicobacter pylori, is there any data in the studies? If not, please add a note in the limitations.

**Response #6:** Thank you for your insightful comment regarding Helicobacter pylori. While H. pylori infection is indeed a significant factor in many upper gastrointestinal disorders, particularly gastritis and peptic ulcer disease, our study did not specifically analyze data related to H. pylori due to a lack of consistent reporting across the included studies. We agree that this is an important limitation, and we have added a note to this effect in the limitations section of the manuscript as follows. “Moreover this study did not account for H. pylori infection due to inconsistent reporting across the studies included in our analysis. As H. pylori is a key factor in several upper gastrointestinal diseases, future research should aim to address this gap by including data on H. pylori status to provide a more comprehensive understanding of its role.”

**Comment #7:** Much of the discussion seems to repeat the results. The focus should be on discussing the findings. Why are the findings in Africa different from other regions in the world? Consider lifestyle, diet, chronic diseases like diabetes and obesity...and other causes. For example, reflux is lower in Africa. I would suggest including more studies from the Middle East, which is close to Africa, if available. Here are three examples:

Abu-Freha N, Gat R, Philip A, Yousef B, Ben Shoshan L, Yardeni D, Nevo-Shor A, Novack V, Etzion O. Indications and Findings of Upper Endoscopies in Males and Females, Are They the Same or Different? J Clin Med. 2021 Apr 11;10(8):1620. doi: 10.3390/jcm10081620. PMID: 33920408; PMCID: PMC8070302.

Sperber AD, Bangdiwala SI, Drossman DA, Ghoshal UC, Simren M, Tack J, Whitehead WE, Dumitrascu DL, Fang X, Fukudo S, Kellow J, Okeke E, Quigley EMM, Schmulson M, Whorwell P, Archampong T, Adibi P, Andresen V, Benninga MA, Bonaz B, Bor S, Fernandez LB, Choi SC, Corazziari ES, Francisconi C, Hani A, Lazebnik L, Lee YY, Mulak A, Rahman MM, Santos J, Setshedi M, Syam AF, Vanner S, Wong RK, Lopez-Colombo A, Costa V, Dickman R, Kanazawa M, Keshteli AH, Khatun R, Maleki I, Poitras P, Pratap N, Stefanyuk O, Thomson S, Zeevenhooven J, Palsson OS. Worldwide Prevalence and Burden of Functional Gastrointestinal Disorders, Results of Rome Foundation Global Study. Gastroenterology. 2021 Jan;160(1):99-114.e3. doi: 10.1053/j.gastro.2020.04.014. Epub 2020 Apr 12. PMID: 32294476.

Sperber AD, Freud T, Abu-Freha N, Shibli F, Brun R, Bangdiwala SI, Palsson OS, Dickman R. Epidemiology of disorders of Gut-Brain interaction in Israel: Results from the Rome Foundation global epidemiology study. Neurogastroenterol Motil. 2022 Aug;34(8):e14323. doi: 10.1111/nmo.14323. Epub 2022 Jan 24. PMID: 35072332.

**Response #7:** Thank you for your suggestion to include more studies from the Middle East, which we agree would strengthen the discussion, given its proximity to Africa. As per your recommendation, we have incorporated findings from the second paper you mentioned: Sperber AD, Bangdiwala SI, Drossman DA, Ghoshal UC, et al. "Worldwide Prevalence and Burden of Functional Gastrointestinal Disorders, Results of Rome Foundation Global Study" (Gastroenterology, 2021). This study provides valuable insights into functional gastrointestinal disorders globally, including data from the Middle East and Africa, which we have used to enhance the comparative analysis in our discussion.

We have now revised the discussion to highlight regional differences, including those between Africa and the Middle East, while also exploring potential contributing factors such as lifestyle, diet, and chronic diseases. We appreciate your input and believe this addition strengthens the global context of our findings.

**Response to Comments of the Reviewer #3**

**Comment #1:** This is an important article providing valuable information on gastrointestinal diseases in Africa, through a systematic review and meta-analysis conducted using electronic databases such as PubMed, Google Scholar, and Hinari, and manual searches of relevant websites. I believe that your article if published, will make positive contributions to the medical literature.

**Response #1:** The authors thank the reviewer for this kind deep appreciation. We greatly appreciate your recognition of the values that our study brings to the medical literature, particularly regarding gastrointestinal disease in Africa. We are committed to ensuring that the final manuscript reflects the highest standard of rigor and clarity.

**Response to Comments of the Reviewer #4**

**Comment #1:** The scope of data taken is too broad, so the results are not specific. There are overlapping indications, for example, dyspepsia, early satiety, anorexia, bloating, postprandial fullness, vomiting, and belching. I evaluate this article that there is nothing we can learn from this paper.

**Response #1:** Thank you for your feedback. We understand your concerns about the scope of the data and the potential overlap of certain indications. However, our objective was to provide a comprehensive analysis of the most commonly reported symptoms in patients undergoing upper gastrointestinal endoscopy (UGIE) across Africa. We extracted data directly from the studies as reported and avoided reclassifying or merging indications to ensure consistency in how each study presented its findings. We acknowledge that some of these symptoms, such as dyspepsia, early satiety, and bloating, may indeed overlap in clinical presentation. However, the broad scope allows for an in-depth view of the diversity in patient presentations, which is important in a large-scale review of multiple studies across a continent with varying healthcare settings. We also recognize that narrowing the focus could provide more specificity. Your feedback is valuable, and we have carefully considered it to improve the interpretation of our findings.
