# Supplementary material for: Indications and endoscopic findings of upper gastrointestinal diseases in Africa: A systematic review & meta-analysis
Source: PLoS One. 2025 Mar 13;20(3):e0319854. doi: 10.1371/journal.pone.0319854 (PMC11906052; doi:10.1371/journal.pone.0319854)
Supplement: Attachment — Submitted filename: Response to Rivewers.docx [file pone.0319854.s011.docx]

**A Rebuttal Letter**

Dear esteemed editor **Dr. Muhammad Salman Bashir** and reviewers,

We greatly value your feedback and comments provided on our manuscript titled "Indications and Endoscopic Findings of Upper Gastrointestinal Diseases in Africa: A Systematic Review & Meta-analysis". All of the points that you raised are important, improve the quality of the work, and contain details that the journal requires.

Dear, we made every effort to answer every one of your remarks. The responses that follow have been made in response to each one. Please find below a point-by-point response to all comments and concerns raised. Reviewer comments are shown in black, followed by our corresponding responses in dark blue. Changes to the manuscript have been highlighted in blue in the revised version for easy access. We want to thank you again for your wonderful feedback and appreciate any more suggestions or remarks you may have to help us improve the work.

Best regards,

A point-by-point response to the comments given

A. **Response to Comments of the Academic Editor**

Dear Dr. Abdu,
Thank you for submitting your manuscript to PLOS ONE. After careful consideration, we feel that it has merit but does not fully meet PLOS ONE’s publication criteria as it currently stands. Therefore, we invite you to submit a revised version of the manuscript that addresses the points raised during the review process.
Please submit your revised manuscript by Jan 31 2025 11:59PM. If you will need more time than this to complete your revisions, please reply to this message or contact the journal office at [plosone@plos.org](mailto:plosone@plos.org). When you're ready to submit your revision, log on to <https://www.editorialmanager.com/pone/> and select the 'Submissions Needing Revision' folder to locate your manuscript file.
Please include the following items when submitting your revised manuscript:

- A rebuttal letter that responds to each point raised by the academic editor and reviewer(s). You should upload this letter as a separate file labeled 'Response to Reviewers'.
- A marked-up copy of your manuscript that highlights changes made to the original version. You should upload this as a separate file labeled 'Revised Manuscript with Track Changes'.
- An unmarked version of your revised paper without tracked changes. You should upload this as a separate file labeled 'Manuscript'.

If you would like to make changes to your financial disclosure, please include your updated statement in your cover letter. Guidelines for resubmitting your figure files are available below the reviewer comments at the end of this letter.

If applicable, we recommend that you deposit your laboratory protocols in [protocols.io](http://protocols.io/) to enhance the reproducibility of your results. Protocols.io assigns your protocol its own identifier (DOI) so that it can be cited independently in the future. For instructions see: [https://journals.plos.org/plosone/s/submission-guidelines#loc-laboratory-protocols](https://track.editorialmanager.com/CL0/https:%2F%2Fjournals.plos.org%2Fplosone%2Fs%2Fsubmission-guidelines%23loc-laboratory-protocols/1/010f0193d6fa15fa-1a9467b3-7239-417a-b479-e748ac049eff-000000/s-3aalFy1TIT2QBniSXVBu2ZvGbCYFKsCsjqBSAgMFk=189). Additionally, PLOS ONE offers an option for publishing peer-reviewed Lab Protocol articles, which describe protocols hosted on [protocols.io](http://protocols.io/). Read more information on sharing protocols at [https://plos.org/protocols?utm_medium=editorial-email&utm_source=authorletters&utm_campaign=protocols](https://track.editorialmanager.com/CL0/https:%2F%2Fplos.org%2Fprotocols%3Futm_medium=editorial-email%26utm_source=authorletters%26utm_campaign=protocols/1/010f0193d6fa15fa-1a9467b3-7239-417a-b479-e748ac049eff-000000/SSFF-7MqYsAKlciAsPsBvjtJDGhfjjypD-D0zugh77o=189).
We look forward to receiving your revised manuscript.

Kind regards,

Muhammad Salman Bashir, M.S.C
Academic Editor
PLOS ONE

[Note: HTML markup is below. Please do not edit.]
Reviewers' comments:
Reviewer's Responses to Questions

**Comments to the Author**

1. If the authors have adequately addressed your comments raised in a previous round of review and you feel that this manuscript is now acceptable for publication, you may indicate that here to bypass the “Comments to the Author” section, enter your conflict of interest statement in the “Confidential to Editor” section, and submit your "Accept" recommendation.

Reviewer #3: All comments have been addressed

Reviewer #5: All comments have been addressed

Reviewer #6: (No Response)

Reviewer #7: (No Response)

2. Is the manuscript technically sound, and do the data support the conclusions?
The manuscript must describe a technically sound piece of scientific research with data that supports the conclusions. Experiments must have been conducted rigorously, with appropriate controls, replication, and sample sizes. The conclusions must be drawn appropriately based on the data presented.

Reviewer #3: Yes

Reviewer #5: Yes

Reviewer #6: Yes

Reviewer #7: Partly

**Response:** Thank you for your comments on the manuscript. I appreciate your insights and recognize the importance of ensuring that the research is technically sound and that the data fully supports the conclusions. I have addressed the points you raised below to clarify and strengthen the manuscript:

3. Has the statistical analysis been performed appropriately and rigorously?

Reviewer #3: Yes

Reviewer #5: Yes

Reviewer #6: Yes

**Response:** Reviewers #3, #5, and #6. Thank you for your positive assessment regarding the statistical analysis. I appreciate your acknowledgment of the rigor and appropriateness of the methods employed in this study**.**

**Reviewer #7: I don’t Know**

**Response:** Thank you for your comment regarding the statistical analysis. I understand the importance of ensuring that the methods used are clearly described and rigorously applied. The statistical analysis was conducted using Stata version 17, a reliable and widely used tool for meta-analysis and statistical modeling. Forest plots were generated using Stata as supporting information to visually present the pooled estimates and heterogeneity across studies. Additionally, the extracted dataset was provided to the journal as supplementary material for transparency and to facilitate reproducibility.

4. Have the authors made all data underlying the findings in their manuscript fully available?

The [PLOS Data policy](http://track.editorialmanager.com/CL0/http:%2F%2Fwww.plosone.org%2Fstatic%2Fpolicies.action%23sharing/1/010f0193d6fa15fa-1a9467b3-7239-417a-b479-e748ac049eff-000000/nznisTfsSZd3YHnc9nw09eSyZLAnN0ZxlxJfw6WuuZQ=189) requires authors to make all data underlying the findings described in their manuscript fully available without restriction, with rare exception (please refer to the Data Availability Statement in the manuscript PDF file). The data should be provided as part of the manuscript or its supporting information, or deposited to a public repository. For example, in addition to summary statistics, the data points behind means, medians and variance measures should be available. If there are restrictions on publicly sharing data—e.g. participant privacy or use of data from a third party—those must be specified.

Reviewer #3: Yes

Reviewer #5: Yes

Reviewer #6: Yes

Reviewer #7: Yes

**Response:** Reviewers #3, #5, #6, and #7 Thank you for your positive assessment regarding the data availability in our manuscript. We are pleased to confirm that all data underlying the findings have been made fully available, in compliance with the PLOS Data Policy.

5. Is the manuscript presented in an intelligible fashion and written in standard English?

PLOS ONE does not copyedit accepted manuscripts, so the language in submitted articles must be clear, correct, and unambiguous. Any typographical or grammatical errors should be corrected at revision, so please note any specific errors here.

Reviewer #3: Yes

Reviewer #5: Yes

Reviewer #6: Yes

**Response:** Reviewers #3, #5, and #6 thank you for your positive remarks regarding the clarity and quality of the manuscript's language. We are grateful for your recognition that the manuscript is written in Standard English and presented in an intelligible manner.

Reviewer #7: No

**Response:** Thank you for raising concerns regarding the manuscript's language. We recognize the importance of presenting the work clearly and ensuring it adheres to the highest linguistic standards. To address your concerns, we have thoroughly reviewed the manuscript to correct any typographical or grammatical errors and made necessary revisions. The language has been refined for better clarity, coherence, and precision to ensure it meets the required standard.

6. Review Comments to the Author
Please use the space provided to explain your answers to the questions above. You may also include additional comments for the author, including concerns about dual publication, research ethics, or publication ethics. (Please upload your review as an attachment if it exceeds 20,000 characters)

**Reviewer #3:** Dear Author,
I have previously evaluated the article titled "Indications and Endoscopic findings of upper gastrointestinal diseases in Africa: A systematic Review & Meta-analysis" with the number PONE-D-24-29678R1 and stated that its publication in your journal would contribute positively to the medical literature. I have also reviewed the criticisms of other referees and the authors' responses to the criticisms regarding the article sent to me for re-evaluation. I believe that all the corrections made by the authors after the criticisms of other referees are sufficient. In my opinion, the article would be appropriate to publish.
Best regards.

**Response:** Thank you very much for your kind and thoughtful evaluation of our manuscript. We truly appreciate your ongoing support and recognition of the contributions our article can make to the medical literature. We are grateful for your review of the revisions we made in response to the criticisms from other reviewers. It is reassuring to hear that you find the changes sufficient and that you consider the article appropriate for publication. Your positive assessment is greatly appreciated, and we remain committed to ensuring the quality and clarity of our work.

**Reviewer #5:** The authors have responded adequately to the review comments, addressing most of the concerns thoroughly. While there remains an unresolved point regarding the lack of specific data on the overall number of upper GI endoscopy cases in Africa and the study's proportionate representation within that total (Comment #6), the authors have explained that a comprehensive data source for all UGIE cases across Africa is unavailable. Given this limitation, the authors have taken steps to mitigate representational bias by including a geographically diverse range of studies and acknowledging this limitation in the revised manuscript.
The remaining unresolved point is noted as a future consideration and does not detract from the overall adequacy of the authors' responses. The reviewers find the authors' explanations and the additional transparency measures, including the full search strategy and supporting data files, sufficient to support the credibility and generalizability of the study's findings.

**Response:** Thank you for your valuable comments and recognition of our effort and for bringing attention to the concern, regarding the lack of specific data on the total number of upper gastrointestinal endoscopy (UGIE) cases in Africa. We would like to clarify that, of the total sample size of 120460 UGIE cases, we identified above 78% of those cases had at least one pathological findings in the included studies. From this subset, we identified 31 common endoscopic findings, including gastritis, esophageal cancer, peptic ulcer disease (PUD), and others. This information has been clearly presented in the Results section (Table 3 and 4), as well as discussed in discussion section. We have taken steps to reduce representational bias by including studies from a geographically diverse range of regions across Africa, despite the absence of a comprehensive data source for all UGIE cases. We acknowledge this limitation in the manuscript and have highlighted it for future consideration. We hope this clarification provides a comprehensive response to your concern and appreciate your recognition of our efforts to ensure the credibility and generalizability of our findings.

**Reviewer #6:** I have been included as a reviewer after some comprehensive recommendations were made by others and read the latest corrected text.

**Response:** Thank you for your valuable time and for reviewing the revised manuscript. We appreciate your consideration of the corrected text, and we are grateful for the comprehensive recommendations made by previous reviewers. Your feedback, along with the contributions of others, has been instrumental in enhancing the quality and clarity of our work

This systematic review and meta-analysis, while comprehensive, does have limitations that may affect the generalizability of the findings. 1) The reliance on institutional samples could lead to an overestimation of the prevalence of UGIE indications and findings, as these samples often consist of patients presenting with severe, persistent symptoms or advanced age. 2) The potential for publication bias should also be noted; studies reporting significant or positive results are more likely to be published, which may skew the pooled prevalence rates reported in the analysis. 3) Additionally, the restriction to English-language publications may have resulted in the exclusion of relevant data from non-English studies, further affecting the comprehensiveness of the findings.

**Response:** Thank you for your thoughtful comments. We appreciate your recognition of the comprehensiveness of our systematic review and meta-analysis. We also acknowledge the important limitations you have raised, which are relevant to the generalizability of the findings.

**Reliance on Institutional Samples**: We agree that the use of institutional samples may introduce bias, particularly by over representing cases of upper gastrointestinal disease. We have recognized this limitation in the manuscript and have made it clear that the findings may not fully reflect the broader population of upper gastrointestinal disease patients, especially those with rural, or from primary care settings.

**Publication Bias**: We also acknowledge the potential for publication bias, as studies with significant or positive results are more likely to be published. This limitation is noted in the manuscript, and we emphasize that the pooled prevalence rates may be influenced by this bias, affecting the overall validity of our estimates.

**Restriction to English-Language Publications:** We recognize that restricting the review to English-language publications may exclude relevant data from non-English studies. This is indeed a limitation of the review process, and we have transparently stated this in the manuscript to highlight the potential impact on the comprehensiveness of our findings.

4)Moreover, Africa is a huge continent where life style, socioeconomics or eating habits varies from north to south + east to west. Instead of generalisation of the whole continent data, it would be better if authors would be able to focus on regional findings. However, the solution of this problem will bring some workload as further dataset analysis that the authors may not prefer.

**Response:** Thank you for your insightful comment regarding the diversity within Africa and the suggestion to focus on regional findings. We agree that Africa is a vast continent with significant variations in lifestyle, socioeconomics, and dietary habits from region to region. In response to your suggestion, we have made an effort to analyze regional differences in the data where possible and have included regional distribution in our result by adding new table 4, and made discussion about the regional distribution variation.

5) The discussion addresses the frequency of all symptoms and outcomes and compares them as numbers. However, does not discuss substantially the possible causes that those differences arise.

**Response:** Thank you for your comment regarding the discussion of the frequencies of symptoms and outcomes. We appreciate your suggestion to delve deeper into the possible causes behind the observed differences. In response, we have expanded the discussion to include potential factors that may contribute to the variations, such as regional differences in healthcare access, socio-economic factors, lifestyle, dietary habits, and other underlying medical conditions that could influence the prevalence and presentation of symptoms.

**Reviewer #7:** Reviewer Comments for: Indications and Endoscopic Findings of Upper Gastrointestinal Diseases in Africa – A systematic Review and Meta-analysis - PONE-D-24-29678R1
1. The abstract should be re-written based on changes suggested below.

**Response:** Thank you for your suggestion regarding the abstract. We have revised it to reflect the changes made throughout the manuscript, including the adjustments based on your comments. The updated abstract now provides a clearer summary of the key findings and addresses all the relevant points as discussed in the manuscript.
2. The introduction section does not adequately explain why it was important to review published reports of indications for, and findings at upper gastrointestinal endoscopy (UGIE) in Africa over a 20-year period. Merely reviewing the pooled prevalence of indications and endoscopic findings in Africa, as the authors have done, is, in my opinion, not enough. Key issues to bring forward here are that these indications/findings may vary across the continent, may change over time and may differ according to age and gender. The main purpose of the review, then, should be to help further understanding of upper gastroduodenal diseases in Africa and to see how they compare to the rest of the world.

**Response:** Thank you for your insightful comments regarding the introduction section of our manuscript. We greatly appreciate your emphasis on the importance of considering the variability in upper gastrointestinal endoscopy (UGIE) indications and findings across Africa, as well as the need to contextualize these findings in terms of geographical, temporal, and demographic factors. In response to your comments, we have revised the introduction to emphasize the fact that UGIE indications and findings may differ across Africa due to diverse socio-economic, healthcare, and environmental factors. This regional variation is crucial for understanding the broader implications of UGID in different contexts. Moreover, we acknowledge that UGID patterns may change over time, influenced by shifts in lifestyle, diet, and access to healthcare. Understanding these temporal trends is vital for guiding public health policies and interventions. Finally in addition to pooling the prevalence data, we have clarified that the main purpose of our review is to enhance understanding of UGID in Africa, compare the findings with global data, and provide insight into how regional, and temporal. These revisions aim to provide a more comprehensive explanation of why this review is essential, beyond simply aggregating the prevalence of UGID indications and findings.

a) The authors should define what they mean by “indications” and “findings” based on standard criteria. The sentence on line 31 and 33 lists indications for UGIE but is inappropriately referenced: Ref 7 (Almario et al., 2018) does not even mention the word “endoscopy” in the entire manuscript while ref 10 is an incomplete book reference. A more suitable recent reference for indications for UGIE would be this: ASGE Standards of Practice Committee et al. Appropriate use of GI endoscopy. Gastrointest Endosc 2012; 75: 1127 – 31. doi: 10.1016/j.gie.2012.01.011

**Response:** Thank you for your valuable recommendation. We have made the necessary revisions in response to your suggestions. Specifically, we have defined the terms “indications” and “findings” based on standard criteria outlined in the ASGE Standards of Practice Committee’s 2012 publication on the appropriate use of GI endoscopy. In line with this, we have updated the indications for upper gastrointestinal endoscopy (UGIE) to reflect these standardized recommendations. As you advised, we have removed the references to Almario et al. (2018) (Ref 7) and the incomplete book reference (Ref 10) and replaced them with the more appropriate and recent reference: ASGE Standards of Practice Committee et al. "Appropriate use of GI endoscopy." Gastrointest Endosc 2012; 75: 1127-31.

b) Indications for UGIE appeared not to have been searched enough: only specific findings relating to UGI tract - PUD/Gastroduodenal ulcers/ulcer - appeared to have been searched for. The other possible findings relating to the oesophagus, stomach and duodenum were not searched for at all. (See S1 Appendix).

**Response:** Thank you for touching this important point. The search strategy has been updated to include a broader range of terms covering indications and findings related to the esophagus, stomach, and duodenum. Additional keywords and MeSH terms were incorporated, such as "esophageal diseases," "esophagitis," "gastritis," "gastric cancer," "duodenitis," and others. The databases were re-queried using the expanded search terms to ensure comprehensive coverage. These updates are reflected in the revised manuscript and supplementary material, including S1 Appendix, where the complete search strategy is documented.
c) Many papers, published from Africa, about indications and findings of UGIE were not included in the review. A simple Google Scholar search - indications and upper gastrointestinal endoscopy findings in Africa - will pull up many, many relevant papers, from all regions of the continent and which fulfilled the stated inclusion criteria, were not included in the review. Here's a link of my search: <https://scholar.google.com/scholar?as_ylo=2020&q=Indications+and+upper+gastrointestinal+endoscopy+findings+in+Africa&hl=en&as_sdt=0,5>

**Response:** Thank you for pointing out the possibility of additional relevant papers on indications and findings of UGIE from Africa that were not included in our initial review. We sincerely appreciate your suggestion and the example search link provided, which has been invaluable in refining our methodology.

**Action Taken:** Based on your critical comment, we conducted a comprehensive re-search for studies on UGIE across Africa using Google Scholar. We used the search terms you suggested, along with other appropriate search words for each country in Africa to ensure comprehensive coverage. This effort resulted in the identification of 1,430 potentially relevant papers. Next Steps: We have systematically screened these papers according to our stated inclusion and exclusion criteria. Papers that meet the inclusion criteria was integrated into the review to provide a more thorough and representative analysis of UGIE indications and findings across the continent. We acknowledge that the initial search have not captured all eligible studies, and we regret any oversight. This additional effort demonstrates our commitment to improving the quality and comprehensiveness of our systematic review. We have included a detailed account of this revised search process in the methods section of the manuscript. We sincerely thank the reviewer for their constructive comment, which has significantly strengthened the rigor and robustness of our review.

A simple similar PUBMED search also brought up many African articles not included in the review: <https://pubmed.ncbi.nlm.nih.gov/?term=indications+and+findings+for+upper+gastrointestinal+endoscopy+in+Africa&size=200&filter=dates.2000%2F1%2F1-2024%2F4%2F1>.

**Response:** Thank you for your valuable comment and for providing the PubMed search link. In response, we have revised our search strategy to ensure comprehensiveness and inclusivity. Below are the key updates we made:

**Expanded Search Terms:** We included additional indications and diagnoses from the esophagus, stomach, and duodenum, ensuring a broader coverage of relevant conditions. Examples include: Esophageal cancer, reflux esophagitis, eosinophilic esophagitis, esophageal varices, Barrett's esophagus, esophageal ulcers, and esophageal strictures. Gastric cancer, gastritis, gastric ulcers, gastric erosions, gastric polyps, gastric outlet obstruction, and pyloric stenosis. Duodenal cancer, duodenitis, duodenal ulcers, and duodenal erosions.

**Refined Procedural Keywords**: We expanded procedural terms to include "esophagogastroduodenoscopy," "upper GI endoscopy," and "gastrointestinal endoscopy," ensuring that variations in terminology were captured.

**Geographic Scope**: All African countries were explicitly included in the search to ensure comprehensive representation across the continent. The search was restricted to articles published between January 1, 2000, and December 31, 2024, as per our updated study's timeframe.

The complete and detailed search strategy, including additional keywords and terms, is provided in the Appendix one for transparency and reproducibility. The revised search yielded 241 articles, an increase from the original search results. After screening for relevance based on our predefined inclusion criteria, additional studies were incorporated into the final dataset. These adjustments enhanced the scope and inclusivity of our review, ensuring it aligns with the objectives of the study. We appreciate your suggestion, as it has significantly improved the robustness of our systematic review.
d) Inclusion criteria (line 69): what specific months between 2000 and 2024 was the review based on?

**Response:** Thank you for your question regarding the time frame of the review. Initially, we included studies published between January 2000 and April 2024. However, based on our revised and comprehensive search strategies—including targeted searches through Google Scholar, PubMed, Hinari and search engines like Google and Yahoo—we extended the inclusion period to December 31, 2024. This adjustment ensures that our review is as thorough and up to date as possible. As a result of these efforts, we identified 35 additional studies that met our inclusion criteria. The updated regional distribution of included papers is as follows: Ethiopia (4), Uganda (2), Tanzania (2), Sudan (4), Nigeria (8), Ghana (2), Burkina Faso (1), Ivory Coast (1), Egypt (8), and South Africa (3). To further refine our analysis and minimize selection bias, we re-evaluated the originally included studies and excluded 15 papers that no longer met the updated criteria. These excluded studies were from Cameroon (2), South Africa (2), Morocco (1), Algeria (1), Egypt (2), and Nigeria (2),Mali (1), Libya(1), Ghana(3). These changes reflect our commitment to improving the comprehensiveness and rigor of the review while adhering to our inclusion criteria.

e) Result (Line 93): Caption should be Results not Result

**Response:** Thank you for pointing out the inconsistency in the caption at result. We have corrected "Result" to "Results" to ensure consistency and accuracy throughout the manuscript.

f) Line 97 states 45 publications, instead of 48 included in the analysis

**Response:** Thank you for your valuable observation regarding the discrepancy in the number of publications included in our analysis. Upon revisiting our data and refining our inclusion and exclusion criteria, we identified the need for adjustments to ensure that our review remains thorough and up to date.

After re-evaluating the databases and modifying our criteria, we excluded 15 studies that no longer met the revised inclusion standards. In parallel, our updated search and screening process identified 35 additional studies that met our inclusion criteria. As a result of these revisions, the total number of studies included in our review increased to 68. We believe these modifications enhance the rigor and comprehensiveness of our review and ensure the inclusion of the most relevant and recent evidence available. We have updated the manuscript accordingly to reflect these changes, including the text in line 97 to correctly state that 68 publications were included in the final analysis. Thank you once again for pointing this out, and we appreciate your feedback in improving the quality and clarity of our manuscript.
g) Under characteristics of studies (line 106 to 108), the arithmetic does not add up: 18+13+8+8 = 47 NOT 48

**Response:** Thank you for comment, the discrepancy in the total number of studies reported in the "Characteristics of Studies" section (lines 106–108). After revisiting our dataset and performing a detailed re-search, we have updated the number of studies included in the analysis. Based on the revised dataset, the total number of studies has changed from 48 to 68, reflecting the inclusion of additional studies from our updated search and re-evaluation. We appreciate your attention to detail, and we have ensured that the corrected number of 68 studies is now accurately presented in the revised manuscript.
h) Line 108 – concerning studies conducted in West Africa: 9+5=1= 15 NOT 18. Cameroon and Democratic Republic of Congo are considered Central African countries, not West African.

**Response:** Thank you for pointing out the issue with the regional classification in Line 108. After revisiting our dataset, re-searching the databases, and applying updated inclusion and exclusion criteria, we ensured the accuracy of this classification. This updated analysis reflects the most current and comprehensive evaluation of the included studies, minimizing bias and enhancing the reliability of our findings. Following this reorganization, we included 35 new studies and excluded 15 older studies. The regional distribution of the included studies is as follows: 22 studies were conducted in West Africa, 25 in East Africa, 11 in North Africa, 9 in South Africa, and 1 in Central Africa.
i) Line 110: the authors state: “….additionally, the criteria used to identify UGID were consistent across studies (Table 1)” I do not see any criteria listed in table 1 or any reference provided.

**Response:** Thank you for critical view with the statement regarding the criteria used to identify UGID in Line 110. After reviewing the manuscript, we realized that the criteria were not clearly listed in Table 1, nor was there a reference provided to support the statement. As a result, we have removed this sentence from the paragraph to ensure clarity and accuracy.
4. Comments on Table 1:
a) Countries in the same region not grouped together. This would have been easier on the eye and more logical

**Response:** Thank you for your valuable suggestion regarding the grouping of countries within the same region. We have implemented your recommendation and reorganized the countries to ensure that those within the same region are grouped together. This adjustment enhances the clarity and logical flow of the presentation, making the data easier to interpret.

b) Author-name use inconsistent: In many, surnames and initial(s) are listed while in others, initials come before surnames, while in some, full names are given. In a few listings, only the surname is provided or the first name is given in full, with the surname given as an initial.

**Response:** Thank you for highlighting the inconsistencies in the author-name formatting. We have carefully reviewed and standardized the author-name format throughout the manuscript. In the revised version, we have ensured that the first author's name is provided followed by "et al." for all references, adhering to the appropriate citation style. We appreciate your attention to detail, as this has helped improve the professionalism and consistency of the manuscript.
c) F Ankouane et al. (2015) appears twice and is referenced as nos. 53 and 54. In the reference list, however, 53 is given as Andoulo et al. (2015) and Ankouane et al. (2016). Can the authors resolve this?

**Response:** Thank you for pointing out the discrepancy with the reference for F. Ankouane et al. (2015). We have corrected this issue and ensured that the reference is consistent throughout the manuscript. The correct reference is now included in the reference list, and we have removed any duplicates or inconsistencies related to this citation.
d) A column showing percent male (or female) will be helpful in showing gender differences across studies being reviewed.

**Response:** Thank you for your valuable suggestion regarding the inclusion of sex distribution across studies. In response, we have added two separate columns in the "Characteristics of Included Studies" table, titled "Male (n)" and "Female (n)", to provide the respective numbers of male and female participants for each study where this information was available. We believe this modification adds clarity and improves the presentation of the demographic data in our review. We appreciate your constructive feedback and its contribution to enhancing our manuscript.
e) Most of the studies reviewed were retrospective. It will be nice to show period (years) spanning each study (eg. Ayuo et al.’s study spans 1993 – 2003).

**Response:** Thank you for your insightful comment, regarding the inclusion of study periods. In response, we have added a new column titled **"Years of Span"** to the "Characteristics of Included Studies" table 1. This column provides the year coverage for each study, including examples such as Ayuo et al.’s study spanning 1993–2003. We believe this addition enhances the clarity and depth of our analysis by highlighting the retrospective nature of the studies.
5. Indications for Upper Gastrointestinal endoscopy (line 114):
a. Epigastric pain and dyspepsia are used as separate indications. According to Rome IV criteria, epigastric pain is part of dyspepsia (<https://theromefoundation.org/rome-iv/rome-iv-criteria/>).
b. Most studies on indications and findings of UGI in Africa do not categorize epigastric pain and dyspepsia as separate entities. Additionally, according to the World Endoscopy Organization, dyspepsia is the preferred standard term to refer to symptoms of upper GI disease, which may include epigastric pain, as a reason (indication) for endoscopy. Please see: <https://www.worldendo.org/resources/minimal-standard-terminology>
c. The two conditions should be treated together.

**Response:** We appreciate the reviewer’s insightful comments regarding the categorization of "epigastric pain" and "dyspepsia." In accordance with the Rome IV criteria and the World Endoscopy Organization's Minimal Standard Terminology, we have combined "epigastric pain" and "dyspepsia" into a single indication for upper gastrointestinal endoscopy. As the reviewer correctly noted, dyspepsia encompasses epigastric pain, early satiety, and postprandial fullness. This adjustment aligns with established standards for reporting upper GI symptoms. We have made the necessary revisions in the manuscript, including updating the tables and results, and citing the relevant guidelines from the Rome Foundation and the World Endoscopy Organization to reflect this change. The revised manuscript now treats dyspepsia (which includes epigastric pain, early satiety, and postprandial fullness) as a unified indication for endoscopy.

6. Comment on Table 2.
a. GERD symptoms (third row) are many - including heartburn and regurgitation. Which are the authors referring to: symptoms of GERD or GERD diagnosis? Heartburn is also listed an indication in the table. Indications (reasons) for endoscopy may be symptoms or diseases. Please see: <https://www.worldendo.org/resources/minimal-standard-terminology>

**Response:** Thank you for your valuable comment. After carefully considering your view, we have revised our analysis to clarify that "GERD symptoms" refers specifically to recurrent symptoms including heartburn and regurgitation. These symptoms, which are prominent features of GERD, have been listed under "GERD symptoms" as an indication for UGIE. This revision was made after careful review to avoid any overlap or confusion.
b. Some of the information given is perplexing to me, and I wonder if these are accurate: for example, the indication to rule out cancer of the stomach was reported in only two studies among 4,670 patients. Or take dyspepsia, as an indication: Only about a third of papers (15/48) reported on this indication in about a third of patients (30,105/100,710).

**Response:** Thank you for your thoughtful comments. Upon reviewing our study, we identified 55 studies that used dyspepsia as an indication for upper gastrointestinal endoscopy, which was the highest among all reported indications. We have updated the manuscript accordingly to reflect the more accurate and comprehensive data regarding the prevalence of dyspepsia as an indication.

Regarding the concern about cancer of the stomach, we have replaced the term "rule out cancer of the stomach" with "suspicion of cancer in the stomach," as reported in the original articles. This indication was mentioned in 10 studies. Similarly, for suspicion of cancer in the esophagus, it was reported by 5 studies.
c. Can the authors also show pooled prevalence over time periods, for example, 2000 – 2010 and 2011 – 2024 to see any time trend?

**Response:** Thank you for your suggestion. In response to your request, we have added a new table, Table 4, which presents the pooled prevalence of endoscopic findings and their indications across two time periods: 2000–2010 and 2011–2024.

7. Endoscopic Findings on Upper Gastrointestinal Tract (line 124):
a. Functional dyspepsia is not "a finding" at endoscopy - According to Rome IV criteria, it's the absence of structural disease to explain symptoms. If the authors mean no abnormal finding, they should say so.

**Response:** Thank you for your comment. We have revised the table to reflect the correct terminology, replacing 'functional dyspepsia' with Normal endoscopic findings
b. Result for PUD should also give a breakdown of gastric ulcer (GU), duodenal ulcer (DU), or both in the text for emphasis as such is an important part of the review.

**Response:** Thank you for your comment. We included the breakdown of gastric ulcer (GU), duodenal ulcer (DU), or both in order to provide more detailed and informative insights into the prevalence and distribution of peptic ulcer disease (PUD). We do not have any other specific concerns regarding this breakdown.
8. Comment on Table 3.
a. Port hepatic gastropathy should be portal hypertensive gastropathy

**Response:** Thank you for your valuable view. We have corrected the term 'port hepatic gastropathy' to 'portal hypertensive gastropathy' as recommended.
b. Functional dyspepsia is not an endoscopic finding. Authors to please see the reviewer’s comment above.

**Response:** Thank you for your comment. As per your suggestion, we have corrected the term 'functional dyspepsia' to 'normal endoscopic findings
c. Bile reflex should be bile reflux

**Response:** Thank you for your comment. We have corrected 'bile reflex' to 'bile reflux' as recommended.
d. It will be very informative to provide a column comparing pooled prevalence of endoscopic findings during specific periods of the study. For example, what are the findings during the first part (2000 to 2010) and the later part (2011 - 2024) of the review? This way, readers can see if there’s any change in trend in findings, which the authors should address in the discussion section.

**Response:** Thank you for your valuable suggestion. We have added a new Table 4 to compare the pooled prevalence of endoscopic findings during specific periods (2000–2010 and 2011–2024). This allows for a clearer visualization of any trends or changes over time, which we have also addressed in the discussion section
9. Discussion:
a. General comments:
i. Discussion of the significance of the results of the systematic review inadequate.

**Response:** Thank you for raising this point regarding the significance of the results. We have thoroughly discussed the key findings of our systematic review and meta-analysis in the discussion section, including their implications and relevance to clinical practice and research.
ii. Since this is an African review, I have not seen how the findings have been compared across Africa to see any similarities or differences. Example: is dyspepsia as common indication in East Africa as in West Africa? What are the most common endoscopic findings in each African region? Are they similar or different, and why?

**Response:** Thank you for raising this important point regarding regional comparisons and their implications. In our analysis, we have addressed regional distribution for some aspects, such as the prevalence of indications for upper gastrointestinal endoscopy (UGIE), to provide a clearer picture of variations across Africa. Specifically, we analyzed how common indications such as dyspepsia vary between regions (e.g., East Africa versus West Africa). However, as noted in the limitations section of our manuscript, we did not conduct a detailed analysis linking indications to the most common corresponding endoscopic findings for each indication due to constraints in the availability and uniformity of data across studies. While this would indeed be valuable, the heterogeneity in reporting across studies limited our ability to draw robust region-specific conclusions.

We appreciate your observation, and future research could build on our work by further exploring the relationships between indications and endoscopic findings to provide a more granular understanding of the patterns and potential underlying causes of these differences.
iii. What are the age and gender similarities and differences in indications and findings of upper GI endoscopy in Africa?

**Response:** Thank you for this valuable observation regarding age- and gender-based differences in the indications and findings of upper gastrointestinal endoscopy (UGIE) in Africa. While we recognize the importance of exploring these differences, we were unable to conduct a detailed analysis on age- and gender-specific patterns due to the bulk of data and variability in reporting across the included studies. Many studies either did not stratify their data by age and gender or presented this information in formats that made it challenging to harmonize and include in our meta-analysis.

We have acknowledged this as a limitation in our manuscript, as we agree that understanding such demographic variations would provide a deeper insight into the epidemiology of UGIE findings in Africa. This could be an important avenue for future research, and we believe our study lays a foundation for more focused analyses on this topic.
b. The sentence on lines 144 and 145 states: “This result ranked higher than a population-based study of epigastric pain (21%) conducted in the USA, Canada, and the UK [66].” I do not think this is a valid comparison. Aziz et al.'s study is about a subtype of functional upper gastrointestinal disorder called epigastric pain syndrome, not epigastric pain in general, which may be associated with other upper gastrointestinal diseases including GERD, PUD, gastritis etc.

**Response:** Thank you for your observation regarding the comparison made between our findings and the study by Aziz et al. We have carefully reviewed your comment and acknowledge that Aziz et al.'s study focuses on epigastric pain syndrome as a subtype of functional upper gastrointestinal disorder, which differs from epigastric pain in general. In our revised analysis, we have categorized epigastric pain under dyspepsia, ensuring a more accurate classification and interpretation of our data. This change has addressed the concern, and we have removed the comparison to Aziz et al.'s study to avoid any misrepresentation. We appreciate your input, as it helped us refine and strengthen the accuracy of our analysis and discussion.
c. I don't think the authors have adequately discussed the significance of epigastric pain/dyspepsia as an indication (reason for endoscopy) especially in relation to findings at endoscopy. Is epigastric pain/dyspepsia more likely to be associated with any particular upper gastrointestinal finding at endoscopy?

**Response:** Thank you for raising this important point regarding the significance of epigastric pain/dyspepsia as an indication for endoscopy and its association with specific upper gastrointestinal findings. In the analysis, we categorized epigastric pain under dyspepsia and assessed its overall prevalence as an indication for upper gastrointestinal endoscopy (UGIE). However, due to the heterogeneity and limited availability of studies explicitly linking dyspepsia/epigastric pain with specific endoscopic findings, we did not analyze this association in detail. We acknowledge this as a limitation in our study and have noted that future research could focus on evaluating these relationships to better understand the diagnostic utility of dyspepsia and its subtypes in clinical practice.
d. Authors state in line 154 that “the wide definitions of dyspepsia in the included studies are probably the cause of the high prevalence in our review”. This statement is incorrect to me. Patients complained of symptoms of upper gastrointestinal disease which, collectively, are referred to as dyspepsia. All it means is that it's a common symptom and is not related to any definition.

**Response:** Thank you for pointing out this concern regarding our statement about the wide definitions of dyspepsia. We acknowledge that the original phrasing may have been misleading. Dyspepsia refers to a collection of upper gastrointestinal symptoms, and its high prevalence in our review reflects its commonality as a symptom rather than being influenced by definitional variations. We have revised the statement to clarify this point and ensure it more accurately reflects the nature of dyspepsia as reported in the included studies.

e. Concerning upper gastrointestinal bleeding (line 156), the authors have not adequately discussed the significance of this observation especially as it relates to findings at UGIE. What are the most common findings in patients presenting with hematemesis and melena? Are there age, gender or regional differences in the prevalence? What do these mean?

**Response:** Thank you for your comment regarding the significance of upper gastrointestinal bleeding (UGIB) and its relation to findings at UGIE. In our analysis, we evaluated the regional distribution and trends of UGIB across Africa; however, we did not specifically identify the most common endoscopic findings in patients presenting with hematemesis and melena.

This limitation was primarily due to insufficient stratification and reporting in the included studies, which did not consistently link these specific clinical presentations to their corresponding endoscopic findings. We have acknowledged this as a limitation in the revised manuscript and emphasized the need for future studies to provide more granular data, including associations between UGIB presentations (hematemesis and melena) and specific endoscopic findings, as well as age- and gender-related differences.
f. Reference 67 (Ford et al., 2015) quoted on line 157 (at the end of the sentence on upper gastrointestinal bleeding) is about global prevalence of uninvestigated dyspepsia, nothing to do UGI bleeding mortality.

**Response:** Thank you for pointing out the incorrect citation of Ford et al., 2015, in reference to upper gastrointestinal bleeding (UGIB) on line 157. We acknowledge that this reference pertains to the global prevalence of uninvestigated dyspepsia and is not relevant to UGIB or its associated mortality. In the revised manuscript, we have removed this citation to ensure accuracy and avoid any confusion. We appreciate your careful review, which has helped us improve the clarity and precision of our work.
g. Concerning “functional dyspepsia” (line 173): functional dyspepsia in not an endoscopic finding. If the authors are referring to normal findings at endoscopy, they should say so. They cannot compare this with the global or regional prevalence of functional dyspepsia as defined by Rome IV criteria. Please see: <https://theromefoundation.org/rome-iv/rome-iv-criteria/>

**Response:** Thank you for your comment regarding the term "functional dyspepsia." We have carefully considered your suggestion and have revised the manuscript accordingly. The term "functional dyspepsia" has been replaced with "normal endoscopic findings," as this more accurately reflects the content of our analysis. We have also omitted the comparison with the global or regional prevalence of functional dyspepsia as defined by the Rome IV criteria, as this was not relevant to our study.
h. concerning line 181 – PUD prevalence: the authors have not satisfactorily discussed the significance of their finding. Has there been any change in the pattern of endoscopic finding of PUD (GU and DU) in Africa in the period under a review? What might be responsible for this?

**Response:** Thank you for your insightful comment regarding the prevalence of peptic ulcer disease (PUD) and the significance of our findings. We have thoroughly discussed the patterns of PUD, including gastric ulcer (GU) and duodenal ulcer (DU), and their trends over the period under review. In the revised manuscript, we highlighted changes in the pattern of endoscopic findings, particularly the observed regional differences and shifts in PUD prevalence.
i. Concerning line 186, the prevalence of gastritis: what is the prevalence for gastritis in various regions of Africa according to this review? Is there a change in prevalence of finding of gastritis over 20 years? What is the likely cause? What does it mean?

**Response:** Thank you for your thoughtful comment regarding the prevalence of gastritis and its regional and temporal trends. In the revised manuscript, we have addressed these issues as follows:

**Prevalence across Regions:** We provided a detailed analysis of the prevalence of gastritis across various regions of Africa based on the studies included in our review. The regional distribution has been summarized in the discussion, highlighting variations that reflect differences in population demographics, healthcare access, and diagnostic practices.

**Temporal Trends**: We examined the changes in the prevalence of gastritis over the past 24 years. Our findings suggest that the prevalence has remained relatively stable in some regions but has shown slight variations in others, potentially influenced by changes in Helicobacter pylori infection rates, dietary habits, and increasing healthcare access for endoscopic evaluation.
j. Concerning line 187, the putative high burden of H pylori in Africa: What is this high burden of Helicobacter pylori infection across Africa? How is it different in various African sub-regions? Is H pylori infection rising or declining or stable?

**Response:** Thank you for your comment regarding the high burden of Helicobacter pylori (H. pylori) infection in Africa. While our study did not directly analyze the prevalence or trends of H. pylori infection, we referenced existing literature indicating that Africa carries the highest burden globally. Several studies, both older and more recent, consistently report that the prevalence of H. pylori infection is particularly high in the Western African region, with some variability across other sub-regions.

In the revised manuscript, we have clarified that this observation is based on external literature and not on a direct analysis from our dataset. We have also acknowledged the lack of specific H. pylori data as a limitation of our study, highlighting the need for further research to better understand its distribution and temporal trends across African sub-regions.
k. Concerning line 192, the authors should discuss regional African differences of gastric cancer and how this pooled prevalence compares to the rest of the world.

**Response:** Thank you for your comment regarding the regional differences in gastric cancer prevalence across Africa. In the revised manuscript, we discussed the distribution of gastric cancer across the continent, noting that it follows a relatively normal distribution, without any particular region showing significantly higher rates. This trend has remained consistent over time, with no major shifts observed in gastric cancer prevalence across the African sub-regions. Additionally, we compared the prevalence of gastric cancer in Africa to global burden, noting that while Africa's burden is lower than in high-incidence regions like East Asia, the overall trend is consistent with a normal distribution across the continent.

l. Concerning lines 193 – 201, I really don’t understand the point(s) the authors are making. Their review is about indications (reasons why people are requested to do endoscopy) and findings (what the examination discovers). Endoscopies are in Africa are usually done in hospitals (“institutions”), so I don’t see how reviewing this information overstates anything. If the authors are implying that findings at endoscopy are not a true reflection of upper gastroduodenal diseases in Africa, they should say so.

**Response:** Thank you for your insightful suggestion regarding lines 193–201. We have carefully reconsidered the statement and revised it for clarity in the manuscript. The intention was not to overstate the role of endoscopy but to emphasize the limitations in generalizing the findings, given that endoscopy is typically performed in hospital settings. As such, the findings may not fully represent the overall prevalence of upper gastroduodenal diseases in the general population, especially in regions with limited healthcare access. In the revised manuscript, we have modified the statement to make it clearer that while endoscopy findings are valuable, they may not always provide a comprehensive picture of the true burden of upper gastrointestinal diseases across the continent due to differences in healthcare access, particularly in rural or underserved areas.

10. Conclusion: This should be re-rewritten after all the issues brought up above have been fully addressed.

**Response:** Thank you for your comment regarding the conclusion. We have revised the conclusion after addressing all the issues raised throughout the manuscript. The revised conclusion now better reflects the key findings of the study, including the regional variations, temporal trends, and the implications of the results. We have also ensured that it aligns with the updated discussions and clarifications made throughout the manuscript.
11. References:
a. Referencing style of journal not adhered to - references with multiple (presumably more than 6) authors are merely given as first author, et al.

**Response:** Thank you for pointing this out. We apologize for the oversight regarding the referencing style. As per the journal's guidelines, references with more than six authors should indeed be listed as the first author followed by "et al." We have thoroughly reviewed all references and ensured that they now conform to the required format. Specifically, references with more than six authors have been revised accordingly.
b. Some references not complete – see Ref 10 – Long DL, Harrison’s principles of internal medicine. 2012.

**Response:** Thank you for pointing out the incompleteness of Reference 10. We have corrected it to include all necessary details in accordance with the journal’s referencing guidelines. The updated reference is as follows:

Longo, D. L., Fauci, A. S., Kasper, D. L., Hauser, S. L., Jameson, J. L., & Loscalzo, J. (Eds.). (2012). Harrison's Principles of Internal Medicine (18th ed.). McGraw-Hill Education.
c. Many of the references are not appropriate for the claim being made. I have indicated some in my comments above.

**Response:** Thank you for your valuable comments regarding the references used in the manuscript. We have carefully reviewed and updated the references, ensuring that each one appropriately supports the claims made in the manuscript. The references you indicated have been corrected or replaced where necessary to ensure alignment with the evidence presented. We believe that these revisions now strengthen the manuscript and ensure that the claims are well-supported by the appropriate literature.
